# TnpA_REP_ and REP sequences dissemination in bacterial genomes: REP recognition determinants

**DOI:** 10.1093/nar/gkab524

**Published:** 2021-06-23

**Authors:** Alix Corneloup, Anne Caumont-Sarcos, Alain Kamgoue, Brigitte Marty, Phan Thai Nguyen Le, Patricia Siguier, Catherine Guynet, Bao Ton-Hoang

**Affiliations:** Laboratoire de Microbiologie et de Génétique Moléculaires (LMGM), CBI, CNRS, Université Toulouse UPS, Toulouse, France; Laboratoire de Microbiologie et de Génétique Moléculaires (LMGM), CBI, CNRS, Université Toulouse UPS, Toulouse, France; Image Processing Facility, CBI, Toulouse, France; Laboratoire de Microbiologie et de Génétique Moléculaires (LMGM), CBI, CNRS, Université Toulouse UPS, Toulouse, France; Laboratoire de Microbiologie et de Génétique Moléculaires (LMGM), CBI, CNRS, Université Toulouse UPS, Toulouse, France; Laboratoire de Microbiologie et de Génétique Moléculaires (LMGM), CBI, CNRS, Université Toulouse UPS, Toulouse, France; Laboratoire de Microbiologie et de Génétique Moléculaires (LMGM), CBI, CNRS, Université Toulouse UPS, Toulouse, France; Laboratoire de Microbiologie et de Génétique Moléculaires (LMGM), CBI, CNRS, Université Toulouse UPS, Toulouse, France

## Abstract

REP, diverse palindromic DNA sequences found at high copy number in many bacterial genomes, have been attributed important roles in cell physiology but their dissemination mechanisms are poorly understood. They might represent non-autonomous transposable elements mobilizable by TnpA_REP_, the first prokaryotic domesticated transposase associated with REP. TnpA_REP_, fundamentally different from classical transposases, are members of the HuH superfamily and closely related to the transposases of the IS*200*/IS*605* family. We previously showed that *Escherichia coli* TnpA_REP_ processes cognate single stranded REP *in vitro* and that this activity requires the integrity of the REP structure, in particular imperfect palindromes interrupted by a bulge and preceded by a conserved DNA motif. A second group of REPs rather carry perfect palindromes, raising questions about how the latter are recognized by their cognate TnpA_REP_. To get insight into the importance of REP structural and sequence determinants in these two groups, we developed an *in vitro* activity assay coupled to a mutational analysis for three different TnpA_REP_/REP duos via a SELEX approach. We also tackled the question of how the cleavage site is selected. This study revealed that two TnpA_REP_ groups have co-evolved with their cognate REPs and use different strategies to recognize their REP substrates.

## INTRODUCTION

Although bacterial genomes are small and compact compared to their eukaryotic counterparts, they harbor multiple repeated sequences playing various functions (for review see (1,2)). Among them, REP elements (for Repetitive Extragenic Palindrome) are small palindromic sequences of 20–50 nts preceded by a conserved tetranucleotide, most often GTAG. REPs are present in great numbers, mostly in intergenic regions of bacterial genomes: about six hundred in the *Escherichia coli* K12 genome or thousands of copies in some *Pseudomonas* strains. They are often organized in BIMEs (for Bacterial Interspersed Multiple Elements). These structures combine two REPs in inverse orientation, REP and inverted REP (iREP), separated by a variable linker and frequently found as consecutive tandem copies. Various cellular functions have been attributed to REP/BIME in the structuring and plasticity of the genome, or in the regulation of gene expression at transcriptional, post-transcriptional levels, and in the regulation of stress response (3–10).

**Figure 1.**
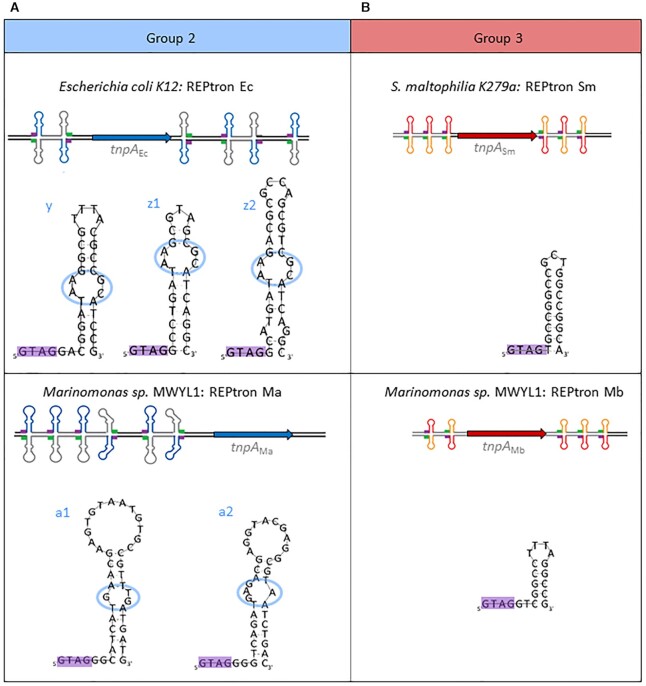
Model REPtrons and corresponding TnpA_REP_/REPs. (**A**) Group 2: *E. coli* MG1655 REPtron Ec, the principal model of the group 2 and three classes of REP y, z1, z2 (top). Analysis of this group was complemented with *in vitro* assays of TnpA_Ec_ on REP_Ma1_ and REP_Ma2_ originated from *Marinomonas* sp. MWYL1 REPtron Ma (bottom). (**B**) Group 3: *S. maltophilia* K279a REPtron Sm (top) and *Marinomonas* sp. MWYL1 REPtron Mb (bottom). In the presented REPtrons, *tnpA*_REP_ (bold arrow) is represented in blue and red in group 2 and 3, respectively. REP and iREP structures are represented in blue/grey (group 2) and red/orange (group 3), respectively, purple and green bold lines—GTAG motif and complementary sequence CTAC, respectively. In the REP detailed structures, the GTAG is boxed in purple. Blue ovals represent irregularities in the group 2 REPs stem. This colour code is maintained throughout the text. For simplicity, in REPtron Ec, y, z1 and z2 REPs are presented without distinction.

A *tnpA_REP_* gene was described to be associated with REPs (11) in its immediate proximity in structures called REPtrons (23) (see examples in Figure 1). For simplicity later on in the text, we will refer to REPtron as to a given encoded protein TnpA_REP_ and the ensemble of cognate REPs. It is important to note that the majority of REPs are generally distributed genome-wide but a given *tnpA_REP_* exists in most cases as a single copy and there is no evidence of *tnpA_REP_* mobility. While the presence of *tnpA_REP_* is often found to be correlated with the abundance of REPs in a given genome (11,12), *tnpA_REP_* behavior, based on several criteria (copy number per replicon, presence on plasmids, duplication rates) resembles more housekeeping genes than transposase genes (13). TnpA_REP_ has thus been proposed to be a domesticated transposase mobilizing REPs over bacterial genomes. However, the underlying dissemination mechanism remains to be elucidated.

TnpA_REP,_ are members of the HuH recombinase superfamily, which includes Rep proteins (rolling circle replication RCR, not to be confused with REP), relaxases (conjugative transfer) and certain Transposases (Helitrons, IS*91*/IS*CR* and IS*200*/IS*605* families). All these proteins cleave, join DNA and carry the characteristic HuH motif (histidine-hydrophobic residue-histidine) crucial for coordinating a metal ion. The metal ion is essential for the nucleophilic attack by the characteristic catalytic Tyr residue, generating a covalent 5′ P-tyrosine intermediate and a free 3′OH after DNA cleavage. Then, the latter 3′OH extremity can serve as primer for RCR, or act as nucleophile to attack the P-tyrosine bond to resolve it (for more details see (14)). TnpA_REP_, while constituting a separate family, are closely related to the transposases of the IS*200*/IS*605* family (TnpA_IS_*_200_*_/IS_*_605_*) of bacterial insertion sequences (IS), which members are bordered by palindromic ends (for review see (15)). Transposition of IS*200*/IS*605* elements occurs on single strand (ss) DNA and is strand-specific (16–18). Moreover, IS*200*/IS*605* cleavage sites are chosen via a peculiar DNA-DNA complementarity between the cleavage sites and the respective ‘guide’ sequences located 5′ to each palindrome (19), (example of model element IS*608* in Supplementary Figure S1).

*tnpA*_REP_ has been found in about 25% of all bacterial species (13). They are present largely in γ-proteobacteria, but also exist in other distant genera. Based on their protein sequences, TnpA_REP_ can be classified into several groups. In particular, groups 2 and 3 (13) (also called groups 2.2 and 2.5, respectively (20)) are associated with the first and best described REPs (7,11,12,21,22). Group 2 mostly includes TnpA_REP_ from different enterobacteria, while group 3 mainly comprises members from *Pseudomonas* species.

These two TnpA_REP_ groups are associated with two types of REPs. Group 2 TnpA_REP_ are associated with long REPs interrupted by an irregular zone/bulge in their stems (Figure 1A, Supplementary Figure S2A top), while group 3 REPs are short and generally perfectly palindromic (Figure 1B, Supplementary Figure S2A bottom). The group 2 TnpA_REP_ from *E. coli* (TnpA_Ec_) is the sole TnpA_REP_ for which experimental studies of interactions with REP substrates have been performed. We have previously shown that TnpA_Ec_ specifically recognizes ss REP (but not iREP) and catalyzes its cleavage and recombination *in vitro*. Cleavage occurs at the dinucleotide CT situated 5′ or 3′ to the REP structure (23). The conserved tetranucleotide GTAG is crucial for this activity. Consistent with this functional role, the GTAG motif forms contacts with several TnpAREP residues, as shown in the co-crystal (24) (see Figure 6B bottom). *E. coli* REPs (REP_Ec_) include two conserved mismatches that form a bulge within the REP stem (Figure 1A). This bulge is required for activity since compensatory mutations restoring regular stem eliminated activity. Although these analyses helped to shed light on the importance of the conserved tetranucleotide GTAG and the bulge in REP_Ec_ recognition by TnpA_Ec_, the role of other components (loop, stem) was still ambiguous. How group 3 TnpA_REP_ recognize their perfect palindromic REPs as well as how the cleavage site is selected remain to be elucidated.

Here, to go further in deciphering TnpA_REP_ activity, we developed a sensitive *in vitro* activity assay, CST (for Cleavage and Strand Transfer) to detect and map REP cleavage sites, that we then adapted to a CST-based SELEX. A combination of this robust approach with a mutational analysis permitted to re-examine and to get access to the importance of different structural features in REP recognition by group 2 TnpA_REP_. In parallel, we extended the analysis to the group 3, for which no data are available, and tackled the question of cleavage site selection in this group. We showed that each group uses different strategies to recognize its REP substrates and demonstrate the role of the GTAG motif in cleavage site selection for a group 3 member. These results represent considerable progress in the comprehension of the distinct mechanism of TnpA_REP_ mediated mobility and specificity of these expanding elements, which led us to discuss REPtrons potential evolutionary routes.

## MATERIALS AND METHODS

### TnpA_REP_ purification

TnpA_Ec_-His6 was purified as previously described (23). TnpA_Mb_ and TnpA_Sm_ coding sequences were synthetized and cloned in suitable expression vector under control of arabinose promotor. TnpA_Mb_ and TnpA_Sm_ were purified by affinity as N-term STREP tag fusion proteins, corresponding proteins were expressed in the *E. coli* K12 Strain Rosetta (DE3) (Novagen). A preculture was grown at 37°C in L broth containing Amp was diluted 50-fold into the same medium at 30°C. Protein expression was induced at OD_600_ = 0.5–0.6 by adding arabinose to 0.8% final. After 3h, bacteria were centrifuged and the pellet was resuspended in buffer NP (phosphate buffer (NaH_2_PO_4_ and Na_2_HPO_4_) pH 8 50 mM, NaCl 400 mM, Triton 0.2%, glycerol 10%, DTT 1 mM) supplemented with 1 mg/ml lysozyme and EDTA-free protease inhibitor cocktail (Roche). Bacteria were sonicated and the lysate was cleared by centrifugation. The supernatant was then mixed with resin Strep-tactin Superflow Plus (Qiagen) during 2h at 4°C. After washes in buffer NP, the proteins were eluted in buffer NPD (phosphate buffer (NaH_2_PO_4_ and Na_2_HPO_4_) pH 8 50 mM, NaCl 400 mM, Triton 0.2%, glycerol 10%, DTT 1 mM, desthiobiotine 2.5 mM). An additional purification step was performed using a Superdex 200 column (Highload 16/60, GE Healthcare). The samples were then dialysed in 25 mM HEPES pH 7.5, 400 mM NaCl, 1 mM EDTA, 1 mM DTT and 20% glycerol and stored at –80°C.

### Standard reactions *in vitro*

Oligonucleotides (Eurofins Genomics) were 5′-end-labelled with [γ-^32^P] ATP (Perkin Elmer) using T4 polynucleotide kinase (Thermo scientific). Labelled oligonucleotides were purified on a G25 column (GE Healthcare).

0.02 μM 5′-labelled oligonucleotide and 0.5 μM unlabelled oligonucleotide were incubated with TnpA_REP_ (45 min, 37°C, final volume 10 μl) in 12.5 mM Tris (pH 7.5), 120 mM NaCl, 5 mM MnCl_2_/MgCl_2_, 1 mM DTT, 20 μg/ml BSA, 0.5 μg of poly-dIdC and 7% glycerol. Reactions were separated on an 8% denaturing gel (7 M urea, Tris Borate EDTA 2 mM, acrylamide 19:1 8%, migration at room temperature at 50 W) and analysed by phosphorimaging. In EMSA experiments, labelled substrates were incubated with corresponding TnpA_REP_ in reaction buffer without divalent metal cation and complexes were separated on 8% native acrylamide gel (Tris acetate EDTA, acrylamide 37.5:1 8%, glycerol 7%, migration at 10 V/cm, 4°C) and analysed by phosphorimaging.

### CST- test on circular substrates *in vitro*

Proteins and substrates were incubated together 45 min at 37°C in the reaction buffer in a final volume of 10 μl containing 50 ng of ∼4kb pBluescript SK- derivative ss phagemid circular substrate, 0.5 μg of poly-dIdC, 1.5 μM TnpA_REP_. 3 μl of 10 μM stock of attacking oligonucleotide B457 were added and incubation continued for 30 min. Reaction was stopped and de-proteinized by adding an equal volume of 25 mM EDTA, 0,6 mg/ml Proteinase K and 2% SDS and incubated for 1 h at 37°C. Products were purified on Promega columns (Wizard SV Gel and PCR) and subsequently served as templates for PCR amplification with GoTaq polymerase using B457 and Cy5 or Cy3 substrate specific fluorescent primer (98°C, 2 min, 30× (98°C 30 s, 56°C 30 s, 72°C 30 s)). PCR products were separated on a 8% native polyacrylamide gel and revealed by scan on GE Healthcare Typhoon Trio Imager.

### CST-based selex

1 μl of 1 μM of degenerate substrates (Eurofins Genomics) was incubated with the corresponding TnpA_REP_ in the standard reactional mixture for 45 min at 37°C. The following steps were as described for CST. Amplification was carried out with 457 or other attacking primers and 321, common for all substrates. After sequencing with 321, ss substrates were prepared for next round by asymetric PCR with Phusion polymerase using 0.1 μM 321 and 10 μM of corresponding attacking primer (98°C 30 s, 45× (98°C 10 s, 56°C 10 s, 72°C 10 s)). The quantification procedure is described in details in Supplementary Materials and in Supplementary Figure S3C.

## RESULTS

### Experimental REPtron models

In this study, we focused on *E. coli* MG1655 REPtron (called Ec) as principal model for the group 2 (Figure 1A, top). *E. coli* MG1655 genome harbors 3 types of REP: y (35nts), z1 (29nts) and z2 (37nts) often combined in BIME as mosaics of y-z1 or y-z2 REPs at multiple loci in the genome (7). The three REP_Ec_ types are imperfect palindromes preceded by the characteristic GTAG and can form stem-loop structures interrupted by a conserved AA-GC mismatch forming a bulge, and certain unpaired bases in the loop. In addition, they share several conserved positions in the stem (Supplementary Figure S2B).

To investigate the group 3 TnpA_REP_/REP, several TnpA_REP_ candidates were tested for their expression and solubility in *E. coli*. We chose *Stenotrophomonas maltophilia* K279a and *Marinomonas sp*. MWYL1 genomes (Figure 1B). Organisms of this group often host several REPtrons and carry hundreds of REPs in their genomes (12). Furthermore, *Stenostrephomonads* are omnipresent environmental bacteria often present in the soil, and *S. maltophilia* is an opportunistic pathogen commonly associated with hospital acquired infections. A phylogenetic analysis of REP distribution in a *S. maltophilia* collection has pointed out a dynamic character of the REP/BIME distribution in these genomes suggesting an ongoing proliferation process (12). We chose to study Sm, one of REPtrons in the *S. maltophilia* K279a strains. REPtron Sm carries perfect palindromes REP (REP_Sm_) of 16 nts interrupted by 3 nts and directly preceded by the conserved tetranucleotide GTAG (Figure 1B).

*Marinomonas* sp. MWYL1 genome carries a group 3 REPtron Mb (Figure 1B, bottom) and also a group 2 REPtron Ma (Figure 1A, bottom and see below). REPtron Mb comprises small perfect palindromic REPs (REP_Mb_) of 10 nts, interrupted by 4 nts and separated by 2 bases to the GTAG tetranucleotide. Interestingly, in contrast to the general genome-wide distribution, for both REPtron Ma and REPtron Mb, a physical association between *tnpA*_REP_ genes and REPs is quite pronounced (11). REP_Ma_ and REP_Mb_ are concentrated in proximity to *tnpA*_Ma_ and *tnpA*_Mb_, suggesting that the arrival of these REPtrons was recent and that the corresponding REP copies have been subsequently multiplied in their vicinity.

We concentrated our analyses principally on REPtrons Ec, Sm and Mb. The three purified TnpA_REP_ were then used to examine their activities on their cognate REPs. The study on the group 2 was also supplemented by activity tests performed with TnpA_Ec_ on group 2 REP_Ma_ from *Marinomonas sp*. MWYL1 genome (Figure 1A, bottom). REPtron Ma includes two types of long REPs (REP_Ma1_ and REP_Ma2_ of 42 and 38 nts) with different irregularities in the stems followed by large loops for which an alignment showed few conserved positions (Supplementary Figure S2C).

### Cleavage and strand transfer assay (CST)

**Figure 2.**
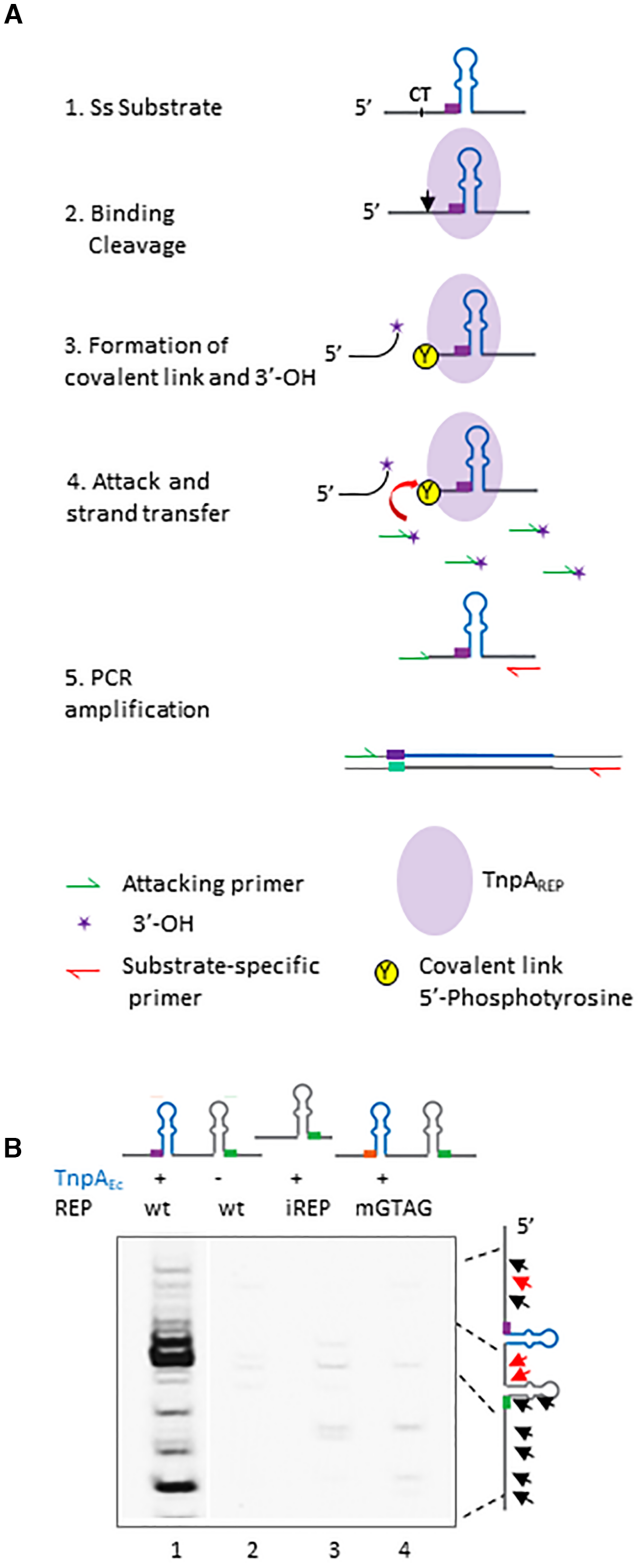
Cleavage Strand Transfer assay (CST). (**A**) Ss DNA substrates (1) were first incubated together with TnpA_REP_ in a reaction buffer leading to their binding and cleavage (2), resulting in the formation of a covalent complex TnpA_REP_ Tyr-5′P and a 3′-OH group (3). Afterwards, an attacking oligonucleotide was added in excess (4), resolving the covalent link and fusing it to the 5′ of cleaved substrate (5). Cleavage sites were mapped by PCR amplification with attacking and substrate-specific primers. Purple oval represents TnpA_REP_, CT/black arrow—cleavage site, purple star - 3′-OH and Y circled in yellow - covalent link Tyr-5′P, respectively. Attacking and substrate specific primers are represented as green and red arrows, respectively. Curved red arrow represents attack by 3′-OH group present on the attacking primer. (**B**) Profile of cleavage sites on ss circular DNA phagemid substrates. The same conditions were used for all the substrates. ‘−’ or ‘+’ indicate no TnpA_Ec_ (lane 2) or with TnpA_Ec_, reactions performed on substrates carrying wild-type REP_Ec_ on a BIME, only iREP or a BIME carrying mutant GTAG (lanes 1–2, 3 and 4 respectively). Black and red arrows (right) represent mapped cleavage sites 5′ and 3′ to REP structure and major cleavage sites in wild-type substrate, respectively.

We previously showed that TnpA_Ec_ is capable of cleaving and recombining ss REP_Ec_*in vitro* (23). Cleavages occur 5′ or 3′ of REP substrates at a dinucleotide C|T. To go further in the comprehension of REP mobility mechanism, we developed an activity assay called CST (Cleavage-Strand Transfer). The CST assay takes advantage of the general property of HuH enzymes, which form a 5′P-tyrosine link and a 3′OH extremity upon cleavage (14) (Figure 2A3). The 3′-OH then can be differently used. Upon cleavage by Rep proteins (single-stranded phages and RCR plasmids) and conjugative relaxases, the 3′-OH group can serve to prime replication. The 3′-OH can also act as the nucleophile for strand transfer to resolve the 5′P-tyrosine link in the termination step of RCR replication, conjugative transfer and transposition. Both possibilities might be exploited to disseminate REP/BIME sequences (23).

The CST assay was first developed with the REPtron Ec (Figure 2). After incubation of ss REP substrates with TnpA_Ec_ in a reaction buffer allowing cleavages to occur (Figure 2A2-3), an excess of an ‘attacking’ oligonucleotide is added and incubation is continued. The 3′OH end of the ‘attacking’ oligonucleotide can then attack the 5′P-tyrosine covalent link to resolve it. This strand transfer reaction leads to the formation of a new molecule where the attacking oligonucleotide is covalently joined to the cleaved ss REP substrate (Figure 2A4). Pilot experiment with attacking oligonucleotides carrying variable 3′ extremities has shown that the 3′ base is obligatory a C, whereas upstream sequence is less important (not shown). To characterize joint products, purified DNA was used as template for PCR amplification using the attacking oligonucleotide and a primer specific for the REP substrate (Figure 2A5). Typical profile obtained with a ss phagemid substrate carrying a wild-type REP/BIME is shown in Figure 2B, lane 1, compared to that obtained in the absence of TnpA_Ec_ (lane 2). No significant amplification products were observed using substrates carrying only an iREP or mutations in the essential GTAG motif (Figure 2B, lanes 3 and 4 respectively). In all cases, amplification was specific to wild-type REP/BIME substrate and wild-type TnpA_Ec_, in contrast to catalytic mutant TnpA_Ec_ Y115F (not shown).

The assay was further validated by sequencing the amplification products. As was the case for experiments documented previously, cleavage occurred mainly in proximity, 5′ or 3′of the REP structure (Figure 2B, Supplementary Figure S3A). We also observed discrete distant cleavage sites. In addition, the attacking oligonucleotide was systematically abutted to the T of the C|T cleavage sites confirming that the amplification products were all issued from cleavage and strand joining events (not shown).

### CST-based SELEX

To get insight directly into REP structural features potentially important for TnpA_REP_ activity, we took advantage of the CST assay to develop a CST-based SELEX (Systematic Evolution of Ligands by Exponential Enrichment) (25). In contrast to the CST assay described above where phagemid-derived circular ssDNA molecules were used generating multiple cleavage sites 5′ and 3′ to the REP, SELEX substrates are simple oligonucleotides carrying a unique 5′ cleavage site and degenerate zones in the REP defining features (the GTAG motif and the palindrome: bulge, loop). These were incubated with cognate TnpA_REP_ as in the CST assay (Supplementary Figure S3B, R_0_). After the first PCR amplification, bulk amplified products were sequenced with a common substrate-specific primer (first round, Supplementary Figure S3B, R_1_). For the next round, ss substrates were prepared by asymmetric PCR using an excess of attacking oligonucleotide as described in Materials & Methods (Supplementary Figure S3B, R2). In each round, different ‘attacking’ oligonucleotides were used, all carrying a 3′C permitting reconstitution of the cleavage site for the next round. Finally, from sequencing data, enrichment of different bases at a given position were estimated by Enrichment factor E_N,0_, calculated as ratio of fractions of a given base at round R_N_ to that at round R_0_: E_N,0_ = *F*_N_/*F*_0_. Level of selection (*S* for score) at each position was then estimated as the variance of E_N,0_ of all bases: *S* = *V*(E_N,0_). The calculation method is detailed in Supplementary Materials and an example of this analysis is illustrated in Supplementary Figure S3C.

**Figure 3.**
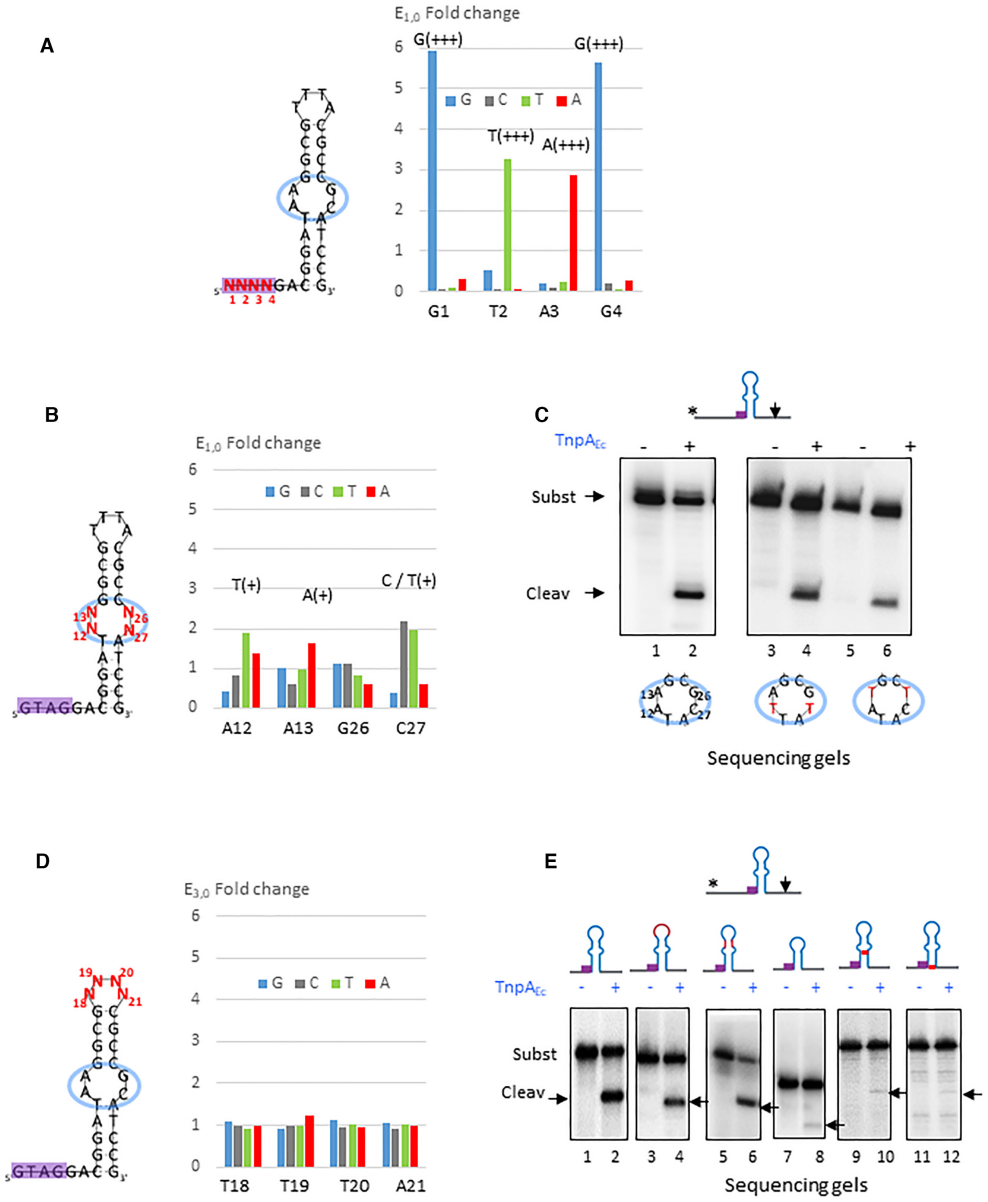
Group 2 *Escherichia coli* REPtron Ec. (**A**) CST-based SELEX and enrichment of the REP_Ec_ GTAG motif. Left: REP_Ec_ structure carrying a 5′ cleavage site (not shown) and degenerate sequence N_1_N_2_N_3_N_4_ (in red) at the GTAG motif. Right: plot representing Enrichment factor E_1,0_ at the first round R1 of the motif at each position with corresponding scores where G, C, T, A are in blue, grey, green and red respectively. Underneath: initial sequence of the motif. (**B**) Importance of the REP_Ec_ bulged region. Left: REP_Ec_ structure carrying degenerate sequence N_12_N_13-_N_26_N_27_ (in red) at the bulged region. Right: plot representing Enrichment factor E_1,0_ of the motif at each position with corresponding scores where G, C, T, A are in blue, grey, green and red, respectively. Below: initial sequence of the motif. (**C**) Cleavage reaction realized with TnpA_Ec_ on wild-type substrate (A_12_A_13_-G_26_C_27_) carrying a 3′ cleavage site (lanes 1–2), substrates carrying T_12_A_13_-G_26_T_27_ (lanes 3–4) and A_12_T_13_-T_26_C_27_ (lanes 5–6) respectively. Below: bulged regions are circled in blue where mutated bases are presented in red and cartoons represent corresponding REP structures. (**D**) SELEX of y REP loop sequence. Left: y REP_Ec_ structure carrying degenerate sequence N_18_N_19_N_20_N_21_ (in red) at the loop. Right: plot representing E_3,0_ at the third round R3 of y REP loop at each position where G, C, T, A are in blue, grey, green and red, respectively. Underneath: initial sequence of the y REP loop. (**E**). Cleavage experiment performed with increasing TnpA_Ec_ on wild-type y REP substrate (lanes 1–2), substrate carrying complement of the loop sequence (lanes 3–4), complement of the superior stem (lanes 5–6), substrate deleted for superior half (lanes 7–8), substrates carrying mutations in the conserved positions T_11_A (lanes 9–10) and G_32_C (lanes 11–12), respectively.

We first tested the CST-based SELEX to re-examine the importance of the conserved GTAG in the REP_Ec_. Supplementary Figure S3C shows sequencing profiles obtained with initial substrate (R_0_) carrying degenerate bases at the GTAG motif and those obtained at the first round (R_1_). Remarkably, the four positions in GTAG motif were selected with high scores at the first round, as illustrated in Figure 3A and Supplementary Figure S3C. This confirmed the crucial role of the motif previously observed: no mutations were tolerated, any substitution abolished binding and cleavage (24, Supplementary Figure S4C lanes 16–18, 19–21 and not shown) and these results therefore validated the test.

### What has been learned from CST-SELEX on the *E. coli* REPtron Ec

We have shown previously that TnpA_Ec_ is active on the three REP_Ec_ y, z1 and z2 (23). In this section, except indicated otherwise, we generally used oligonucleotides substrates carrying derivatives of the y REP_Ec_, the most studied at biochemical and structural levels. REP coordinates were kept as used previously (24).

#### The REP_Ec_ bulge

The conserved mismatches A_12_A_13_-G_26_C_27_ are located in the middle of the y REP stem and the C_27_ base is specifically contacted by TnpA_Ec_ (Figure 6B bottom; (24)). Mutations A_12_A_13_-T_26_T_27_ or G_12_C_13_-G_26_C_27_ introduced to correct the mismatches severely affected activity (24,23). One could therefore expect a significant or exclusive selection of these bases in the CST-based SELEX assay. Instead, while some selections occurred for the three positions A_12_A_13_ and C_27_, the enrichments were far from those observed with the GTAG motif (Figure 3B). In particular, both C and T were only moderately enriched at the C_27_ position which is in contact with the protein. The same was observed with conserved positions A_12_A_13_ where T_12_ and A_13_ were merely enriched with medium scores, respectively (Figure 3B). Medium and low scores could result from the poor selection of independent bases at each position. Alternatively, multiple specific combinations of nucleotides may have been selected. However, bulk Sanger sequencing cannot capture associations between positions and only provide an average picture of the selection process. Since individual selected molecules were not sequenced the analysis cannot inform us directly about synergism or antagonism between substitutions.

To investigate the impact of this ‘low selection’, we tested substrates carrying substitutions A_12_T, C_27_T, replacing natural mismatch positions by those suggested by SELEX or by other bases A_13_T, G_26_T, both keeping bases unpaired. Cleavage of these variants was maintained as judged by the presence of cleavage products (Figure 3C, compare lanes 3–4, 5–6 to 1–2). Thus, the unpaired state (mispairing in this case) instead of the sequence, seems to be crucial for recognition of the REP_Ec_ by TnpA_Ec_.

#### The REP_Ec_ stem-loop

Beyond the conserved bulge, hundreds of y, z1 and z2 REP_Ec_s share several common features (see consensus alignment in Supplementary Figure S2B) including a position in upper stem and several conserved positions in the lower stem, and in particular T_11_ and G_32_ contacted by TnpA_Ec_ (24) (Figure 6B bottom). Among these 3 types of REPs, stem lengths and loop sequences are variable while relatively conserved in each group. To get access to the role of respective loops, we performed CST-SELEX on the 3 types of REPs. No specific enrichment of degenerate loop nucleotides occurred even after several rounds (with E_3,0_ around 1 and low scores for all positions) (Figure 3D, result shown for y and Supplementary Figure S4A-B for z1 and z2 REP_E_c, respectively). We further tested the importance of the upper stem sequence and length by mutations. Binding and cleavage of a P^32^-labelled oligonucleotide substrate for which the loop sequences or the upper stem were swapped to their complement, were still observed, as shown by EMSA (Supplementary Figure S4C, compare lanes 1–3, lanes 4–6 and not shown) and sequencing gel (Figure 3E, lanes 1–2, 3–4 and 5–6), respectively. Similarly, no notable effect was observed upon modification of y REP lower or upper stem to simulate the z1 and z2 structures (not shown). Nevertheless, ablation of the upper stem and loop abolished binding and severely affected cleavage as judged by the absence of retarded complex (Supplementary Figure S4C, lanes 7–9) and reduction of cleavage products (Figure 3E, lanes 7–8). These results suggest a non-specific structural role of the REP_Ec_ upper stem-loop. This is in contrast to the role of the conserved positions T_11_ and G_32_ in the lower stem, which mutations T_11_A or G_32_C seriously affected binding (Supplementary Figure S4C, lanes 10–12 and 13–15, respectively) and cleavage (Figure 3E, lanes 9–10 and 11–12, respectively).

#### Cross-activity

Taken together, these results suggest a relative flexibility in the substrates of TnpA_Ec_. This implies that other REP structures, harboring only few conserved features with REP_Ec_ could be recognized and processed by TnpA_Ec_. Examination of REP structures in two group 2 REPtrons has pointed out some potential common features in REP_Ec_ and REP_Ma_ (Supplementary Figure S5A). Consistently, TnpA_Ec_ exhibits robust activity on REP_Ma1_ and REP_Ma2_ substrates (Supplementary Figure S5B, lanes 1–2 and 3–4, respectively). The importance of the bulge for activity could be confirmed by experiment where mutations introduced to form perfect stem affect TnpA_Ec_ cleavage activity on REP_Ma1_ and REP_Ma2_ substrates (Supplementary Figure S5C, lanes 3–4 compared to 1–2 and lanes 7–8 compared to 5–6).

On top of the crucial GTAG motif, a handful of REP additional structural features appears sufficient to be recognized and processed by TnpA_Ec_.

### *S. maltophilia* REPtron Sm: different strategy to recognize cognate REP

**Figure 4.**
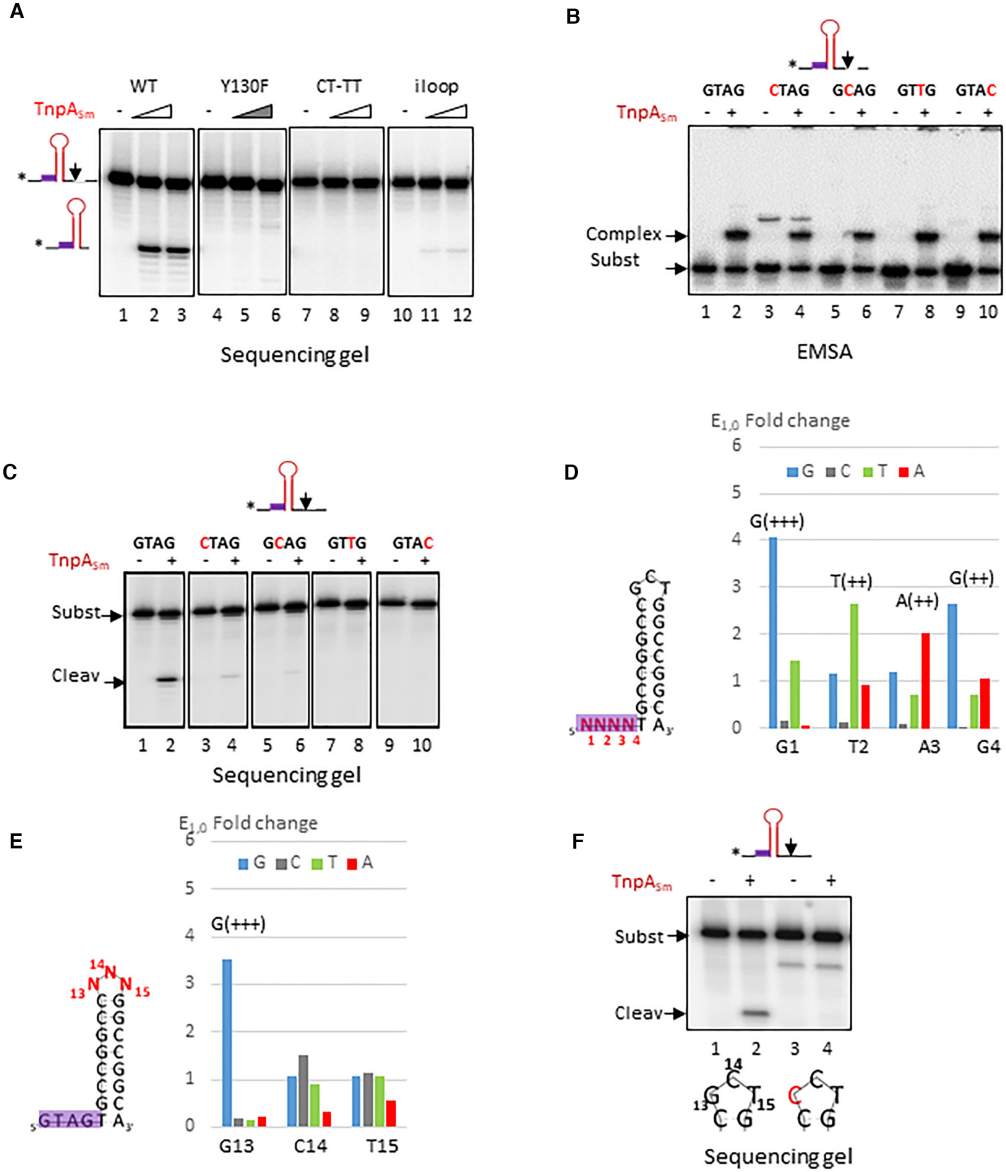
Group 3 *Stenotrophomonas maltophilia* REPtron Sm. (**A**) Cleavage of 55 nts REP_Sm_ substrates performed with increasing concentrations (1 and 4 μM) of wild-type TnpA_Sm_ (lanes 1–3), catalytic mutant derivative TnpA_Sm_ Y130F (lanes 4–6), wild-type TnpA_Sm_ on substrates carrying mutated cleavage site CT-TT and reverse complement of the loop sequence (lanes 7–9 and 10–12, respectively). (**B**) Importance of the GTAG motif for binding. EMSA experiment performed with 2 μM wild-type TnpA_Sm_ on wild-type GTAG substrate (lanes 1–2), CTAG (lanes 3–4), GCAG (lanes 5–6), lanes GTTG (lanes 7–8) and GTAC substrates (lanes 9–10). Mutated positions are indicated in red. (**C**) Importance of the GTAG motif for cleavage. Cleavage experiment performed on a substrate carrying a 3′ cleavage site with 2 μM of wild-type TnpA_Sm_ on wild-type GTAG substrate (lanes 1–2), CTAG (lanes 3–4), GCAG (lanes 5–6), lanes GTTG (lanes 7–8) and GTAC substrates (lanes 9–10). Mutated positions are indicated in red. (**D**) Enrichment of the REP_Sm_ GTAG motif. Left: the same schema as described previously where the REP_Sm,_ structure carrying a 5′ CT cleavage site and a degenerate sequence N_1_N_2_N_3_N_4_ at the GTAG motif. Right: plot representing E_1,0_ (Enrichment factor) of the motif at each position with corresponding scores. Below: initial sequence of the motif. (**E**) REP_Sm_ loop SELEX and enrichment at the first round. Left: REP_Sm_ structure carrying a degenerate sequence N_13_N_14_N_15_ (in red) at the loop. Right: plot representing Enrichment factor E_1,0_ at the first round R1 of the loop at each position where G, C, T, A are in blue, grey, green and red, respectively. High score is indicated for G_13_. Below: initial sequence of the REP_Sm_ loop. (**F**) Effect of loop mutations on activity. Cleavage of 55 nts REP_Sm_ substrate carrying wild-type G_13_C_14_T_15_ or C_13_C_14_T_15_ loop sequence performed with wild-type TnpA_Sm_ (lanes 1–2 and 3–4, respectively).

The REPtron Sm includes REPs of 23 nts (REP_Sm_) composed of an 8-bp perfect palindrome and a 3-nt loop (Figure 1B). Purified Sm TnpA_REP_ (TnpA_Sm_) cleaves REP_Sm_ substrate (an oligonucleotide carrying REP structure and a 3′ cleavage site) at a CT dinucleotide, as shown in Figure 4A (lanes 1–3). No cleavage product was observed with the catalytic mutant derivative TnpA_Sm_ Y130F (lanes 4–6) nor in the presence of a substrate carrying the mutant cleavage site CT-TT (lanes 7–9). As observed for Ec REPtron, the iREP_Sm_ displayed no binding and cleavage activity (not shown). To examine the importance of the conserved GTAG motif and the palindrome features of ss REP_Sm_, we assayed different ss REP_Sm_ substrates for binding, cleavage *in vitro* and SELEX.

#### The GTAG motif

TnpA_Sm_ formed specific retarded complex with ss REP_Sm_, as shown in EMSA experiments (Figure 4B, lanes 1–2). Single mutations in the GTAG motif did not affect the binding profile (Figure 4B, lanes 3–10), showing that, in contrast to TnpAEc (Supplementary Figure S4C, lanes 16–18 and 19–21 and (24)), TnpASm binding to its substrate tolerates mutations in the conserved tetranucleotide. However, these mutations seriously affected cleavage as shown in Figure 4C. Activity was reduced with CTAG mutant and barely detected with GCAG substrate (Figure 4C lanes 3–4 and 5–6, respectively compared to wild-type GTAG, lanes 1–2). Mutations in the third and fourth positions completely abolished cleavage (GTTG, lanes 7–8 and GTAC, lanes 9–10). In agreement with these results, in a SELEX experiment, the GTAG motif was selected was selected mainly with good scores (Figure 4D).

#### The REP_Sm_ stem-loop

In a first series of experiments, we used a mutant carrying a reverse complement of the loop sequence (G_13_C_14_T_15_- A_13_G_14_C_15_). Cleavage was severely affected, as shown in Figure 4A (lanes 10–12). These mutations also largely compromised binding since no retarded complex was observed by EMSA experiment (not shown), suggesting its critical role in REP recognition. We further investigated the importance of the loop by CST-based SELEX (Figure 4E). Among the 3 bases G_13_C_14_T_15_, the G_13_ was largely enriched with high score whereas C_14_ and T_15_ in particular, were not. Accordingly, mutation of a guanine base to a cytosine G_13_C (C_13_C_14_T_15_) abolished cleavage (Figure 4F, compare lanes 1–2 and 3–4), confirming the SELEX result and highlighting the crucial role of this specific position in the REP_Sm_ loop.

To get access to the importance of the REP_Sm_ stem, we introduced mutations mostly by changing nucleotides to their complements by blocs, and subsequently at individual positions. These experiments showed a certain role of the central and upper parts of the stem on cleavage (Supplementary Figure S6A, compare lanes 1–2 with lanes 3–4, 5–6, 9–10, 11–12 and 13–14) although the effect was not drastic. Interestingly, such mutations in three bottom positions improved the cleavage (lanes 7–8). We also tested importance of being a perfect stem by introduction of a mismatch near the middle of the REP_Sm_ stem. These mutations affected or almost eliminated cleavage (Supplementary Figure S6B, compare lanes 4–6, 7–9 to lanes 1–3).

### *Marinomonas* sp. MWYL1 REPtron Mb: ‘flexibility’ in cleavage site selection

**Figure 5.**
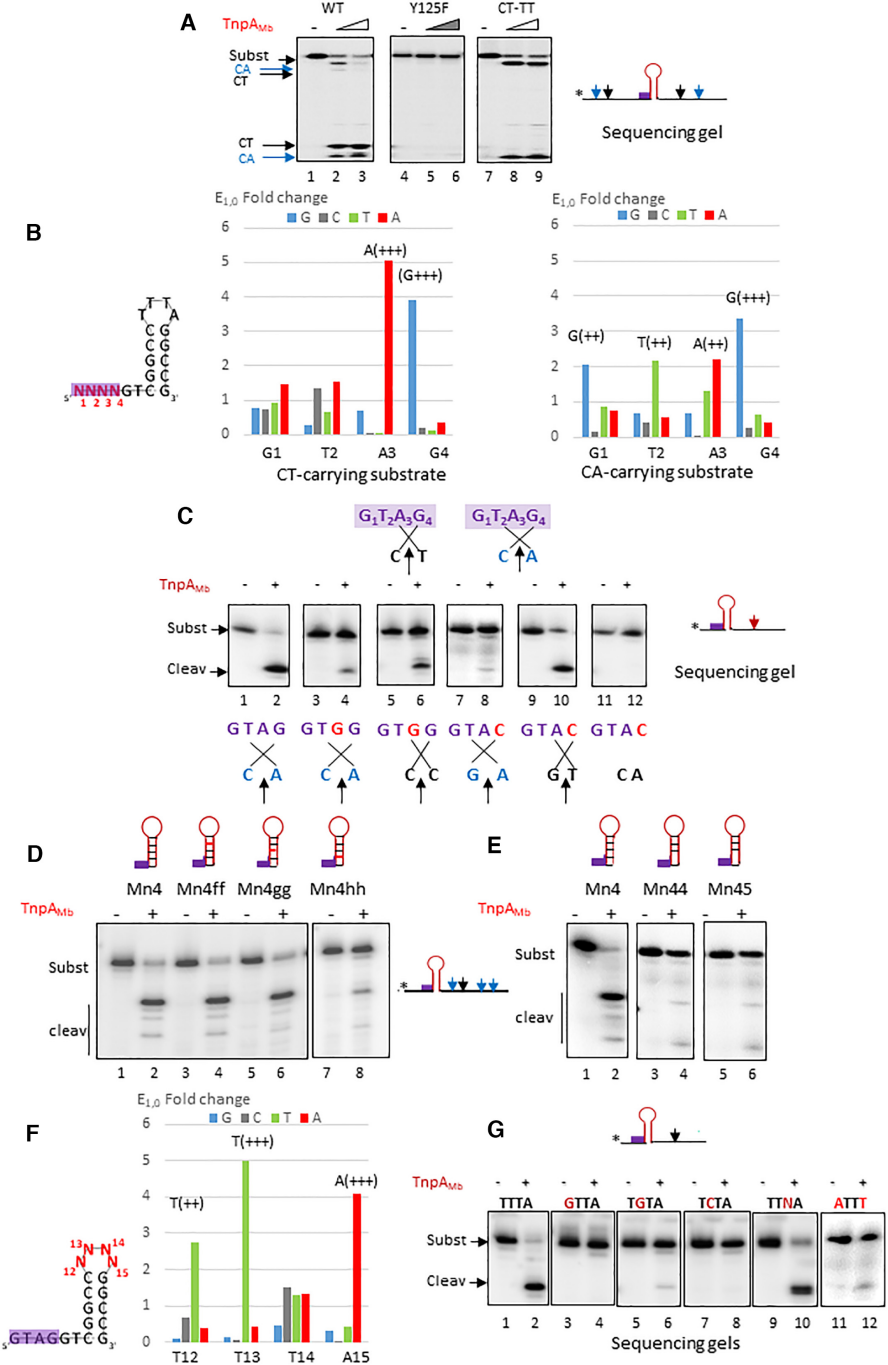
Group 3 *Marinomonas* sp. MWLY1 REPtron Mb. (**A**) TnpA_Mb_ cleaves cognate REP at CT and CA. Cleavage experiment performed with increasing concentrations of wild-type TnpA_Mb_ on 55 nts wild-type substrate (lanes 1–3), TnpA_Mb_ catalytic mutant derivative Y125F on wild-type substrate (lanes 4–6) and substrate carrying mutations CT-TT at two CT sites (lanes 7–9). CA and CT cleavage products are shown by blue and black arrows, respectively. (**B**) REP_Mb_ GTAG SELEX on CT-carrying substrate (left) and on CA-carrying substrate (right). The same schema as described previously is shown, REP_Mb_ carrying a 5′ CT or CA cleavage site and degenerate sequence N_1_N_2_N_3_N_4_ (in red) at the GTAG motif where G, C, T, A are in blue, grey, green and red, respectively. Scores are indicated at corresponding positions. Underneath: initial sequence of the motif. (**C**) Model of CT or CA-cleavage sites selection based on IS*608* model (top). GTAG mutations and cleavage sites selection (bottom). Cleavage of 35 nts simple substrates carrying mutations in the GTAG motif and a 3′ unique cleavage sites: wild-type GTAG and CA (lanes 1–2), GTGG and CA (lanes 3–4), GTGG and CC (lanes 5–6), GTAC and GA (lanes 7–8), GTAC and GT (lanes 9–10), GTAC and CA (lanes 11–12). Schemas of cleavage sites potential selection are shown below each gel. (**D**) Role of REP_Mb_ stem. Cleavage of 39 nts REP_Mb_ wild-type substrate by TnpA_Mb_ (lanes 1–2), substrates with mutated 2nd (lanes 3–4), 3rd (lanes 5–6) and 4th positions (lanes 7–8) respectively. Top: Cartoons representing REP structures with mutated positions in red. (**E**) Role of REP_Mb_ perfect stem. Cleavage of 39 nts REP_Mb_ wild-type substrate by TnpA_Mb_ (lanes 1–2), substrates with mismatch G-A (lanes 3–4) or T-C (lanes 5–6) at 4th position, respectively. Top: Cartoons representing wild-type and REP structures with mutated positions. (**F**). REP_Mb_ loop sequence at R1 selection (E_1,0_) with corresponding scores where G, C, T, A are in blue, grey, green and red, respectively. The same schema as described previously with N_12_N_13_N_14_N_15_ degenerate loop sequence. Underneath: initial sequence of the motif. (**G**) Effect of REP_Mb_ loop mutations on activity. Cleavage experiments on 39 nts simple CT-carrying substrate with wild-type loop T_12_T_13_T_14_A_15_ (lanes 1–2), G_12_T_13_T_14_A_15_ (lanes 3–4), T_12_G_13_T_14_A_15_ (lanes 5–6), T_12_C_13_T_14_A_15_ (lanes 7–8), T_12_T_13_N_14_A_15_ (lanes 9–10) and A_12_T_1_T_14_T_15_ (lanes 11–12), respectively.

The *Marinomonas* group 3 REPtron Mb comprises small 5 bps perfect palindromic REPs, separated by 2 bases from the GTAG tetranucleotide (Figure 1B). Since TnpA_Mb_ binding to its REP substrate cannot be visualized by EMSA probably due to instability of complexes, here we examined only cleavage activity. Interestingly, the system turned out being more flexible: TnpA_Mb_ (Mb TnpA_REP_) cleaved cognate REP_Mb_ at two sites, CT and CA. A ss DNA substrate of 55 nts carrying these cleavage sites both 5′ and 3′ to the stem-loop exhibited 4 cleavage products (Figure 5A, lanes 1–3). Cleavage sites were confirmed by CST assay and mutational analysis. As expected, no cleavage product was observed with the catalytic mutant derivative TnpA_Mb_ Y125F (lanes 4–6). A substrate carrying the mutant cleavage site CT-TT gave rise to cleavage products at the CA sites only (lanes 7–9).

#### The GTAG motif: SELEX and role in cleavage site selection

In the case of REPtron Ec, the GTAG tetranucleotide is not only involved in TnpA_Ec_ recognition of REP_Ec_ but also supposed to participate in cleavage site selection (24). Hence we examine the importance of the GTAG motif by SELEX in oligonucleotide substrates carrying 5′ CT or CA cleavage sites separately. We first observed that the selected profile with a CT-carrying SELEX substrate contrasted with the result obtained with REPtron Ec: only the last two positions were strongly enriched with high scores after a single round of enrichment (Figure 5B, left). The profile obtained with CA-carrying substrate showed mainly moderate, more homogenous selection with relatively good scores for the motif (Figure 5B, right).

Cleavage site of IS*200*/IS*605* family members is selected by particular DNA-DNA linear and cross complementarity with guide sequences, tetranucleotide 5′ to the palindromes at left and right IS ends (19) (Supplementary Figure S1). A simple model of REP cleavage site selection would thus involve the GTAG tetranucleotide as a guide sequence (24). Accordingly, CT and CA can be chosen by cross complementarity with A_3_G_4_ and T_2_G_4_ respectively (Figure 5C, top). To test this hypothesis, we designed simple 38 nts substrates carrying mutated GTAG variants and a unique cleavage site located 3′ to the stem-loop. The wild-type GTAG substrate was cleaved at CA and CT sites (Figure 5C lanes 1–2 and not shown). Although less efficiently, a substrate carrying a mutation of the third base (GTAG-GTGG) was again cleaved at CA, as expected (lanes 3–4). Changing of GTAG to GTGG resulted in cleavage at CC (lanes 5–6) and to GTAC in cleavage at GA (lanes 7–8) and GT sites (lanes 9–10), respectively. Importantly, no cleavage was detected in absence of the corresponding cleavage site (lanes 11–12).

Thus, different positions of the GTAG motif were selected in substrates carrying CT or CA cleavage sites and although efficacy varied, changing a subset of the motif could modify REP_Mb_ cleavage sites in a predictable way according to two presumed schemas and examples shown in Figure 5C. This confirmed the active role of the motif in cleavage sites selection of this ‘flexible’ REPtron, in a manner similar to that described for the IS*200*/IS*605* elements (19,26).

#### The Mb stem–loop

The swap of the entire REP_Mb_ stem to its complement moderately affected cleavage (not shown) indicating a slight role in the REP recognition/activity. Similarly, we further analyzed the importance of different stem portions by the same procedure. We observed a diminution of cleavage activity for mutations of the fourth position in the REP_Mb_ stem, but no effect for mutations of the second and third positions (Figure 5D, compare lanes 1–2 and 7–8 and not shown). Similarly to the REPtron Sm, introduction of a mismatch in the REP_Mb_ stem seriously diminished cleavage activity (Figure 5E, compare lanes 3–4 and 5–6 to lanes 1–2). We then examined the importance of the REP_Mb_ loop by SELEX. Experiments were performed separately on CT- or CA- carrying substrates with a degenerate loop. For both substrates, three among 4 positions (T_12_, T_13_ and A_15_) were strongly enriched with good and excellent scores (Figure 5F and not shown). Accordingly, negative values of log2(E_1,0_) (for E_1,0_ below 1)_,_ clearly illustrated exclusion of the rest in these three positions T_12_, T_13_ and A_15_ (Supplementary Figure S7). These counter-selections were otherwise confirmed by mutational analysis shown in Figure 5G. Mutations in the first and second positions (G_12_T_13_T_14_A_15_ and T_12_G_13_T_14_A_15_) greatly reduced cleavage (Figure 5G, lanes 3–4 and lanes 5–6, compared to lanes 1–2). Also, the replacement T_13_C (T_12_C_13_T_14_A_15_) completely abolished activity (lanes 7–8) while substrate carrying the 14th base degenerate (T_12_T_13_N_14_A_15_) exhibited wild-type behavior (lanes 9–10). Finally, exchange of T_12_ and A_15_ (A_12_T_13_T_14_T_15)_ or individual substitutions T_12_A (A_12_T_13_T_14_A_15)_ or A_15_T (T_12_T_13_T_14_A_15_) also compromised activity, as shown in Figure 5G, lanes 11–12 and not shown.

These results confirmed the crucial role of three positions in the REP_Mb_ loop in cleavage activity and suggest that two bases T_12_ A_15_ are complementary in the REP_Mb_ structure and might be considered as part of the stem.

## DISCUSSION

**Figure 6.**
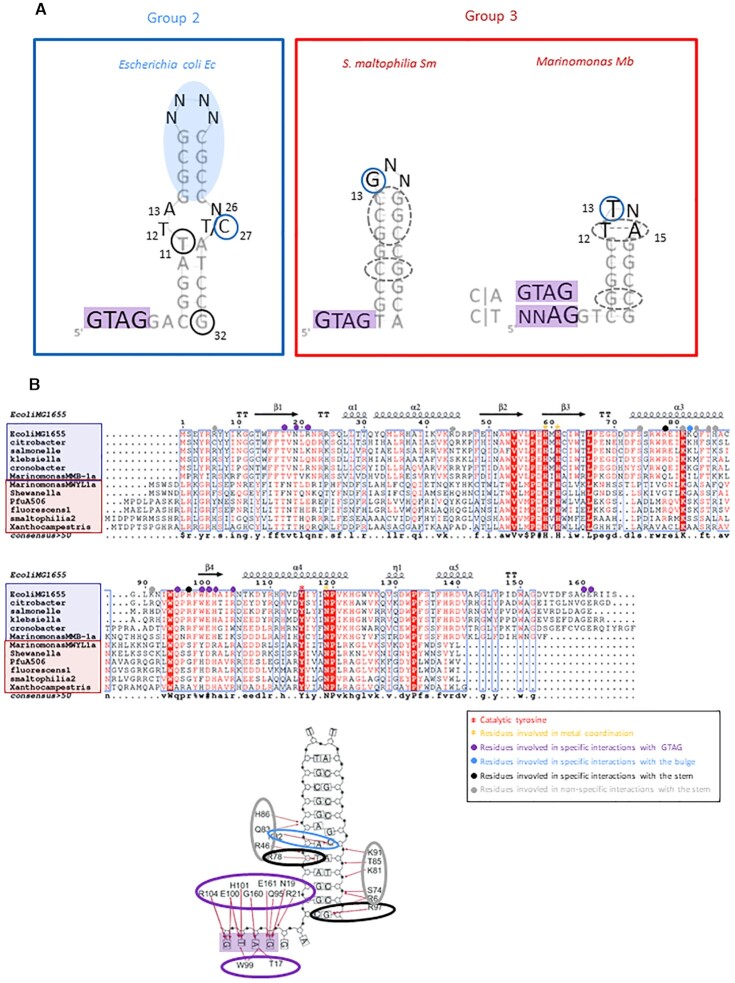
REP structural determinants in three models and putative contacts TnpA_REP_/REP. (**A**) Zones important for TnpA_REP_ activity. Summary of the roles of REP structural components in three models, positions tested by CST-based SELEX are in black, where font size reflects enrichment score. Left: Selected bases in the REP_Ec_ bulge region and non-specific structural upper half (blue oval) is presented. Conserved positions contacted by TnpA_Ec_ C_27_ (bulge) and T_11_, G_32_ (lower stem) are circled in blue and black, respectively, where the latter importance was confirmed experimentally. Right: stem important zones are circled with dotted lines, loop key positions revealed by SELEX are circled in blue. REP_Mb_ GTAG SELEX results of CA and CT-carrying substrates are boxed separately. REP_Mb_ important positions T_12_ and A_15_ are presented paired and circled with dotted lines, as suggested by mutational analysis. (**B**) Top: Alignment of groups 2 and 3 TnpA_REP_ (boxed in blue and red, respectively) based on TnpA_Ec_ structural data. Catalytic tyrosine and HuH motif are indicated by red and orange-coloured stars, respectively. TnpA_Ec_ residues involved in specific contacts with the GTAG motif, specific interaction with the bulge, specific and non-specific contacts with REP_Ec_ stem are indicated by purple, blue, black and grey points, respectively. Bottom: TnpA_Ec_ residues contacting minimal y REP_Ec_ structure (24) where the same colour code is used: residues contacting specifically GTAG (boxed in purple), bulged C_27_ (in light blue) and stem specific positions T_11_, G_32_ (in black) and stem non-specific interactions (in grey), respectively.

Our analysis demonstrated that TnpA_REP_ of the two groups employ diverse strategies to recognize their REP substrates. Clearly both REP components, the GTAG tetranucleotide motif and the palindrome, were involved in TnpA_REP_ activity but their respective impacts varied in each system. In Figure 6A, we summarize the importance of these features. While GTAG is instrumental in REPtron Ec, mutations are largely tolerated in REPtron Sm for binding and in REPtron Mb for cleavage (and by deduction for binding). Although involvement of the GTAG motif in cleavage site selection has been suggested for REPtron Ec, its role was not experimentally supported. Interestingly, the REP_Mb_ tetranucleotide motif was differently selected in CA or CT carrying substrates, probably reflecting their distinct contribution to respective cleavage sites selection. The role of loop sequences is also different for representatives of the two groups. No mutations were tolerated in certain REP_Mb_ or REP_Sm_ loop positions, while only a non-specific structural role was suggested for the REP_Ec_ loop.

### TnpA_REP_ of the two groups

#### Catalytic center and C-term tail

Groups 2 and 3 REPtrons differ by their encoded TnpA_REP_ and corresponding REPs. As shown by an alignment performed on a limited collection of TnpA_REP_ (Figure 6B), the catalytic center composed of the metal coordination module (HuH motif and other additional residues (24)) and the catalytic Tyr is well conserved in both groups. Some differences are found in the N-term and C-term portions: group 3 members include several supplementary residues in N-term whereas group 2 members carry a C-term extension of about 20 residues, comprising a short helix α5 and an unstructured region in the case of TnpA_Ec_ (Figure 6B). The helix α5 and downstream adjacent region appeared to be important in TnpA_Ec_ activity since derivatives Δ131 and Δ144 (deletions of 34 and 21 C-terminal residues, respectively) exhibit serious defects in binding and cleavage (data not shown). However deletion of 13 extreme C-terminal residues resulted in a mutant, Δ152, with higher activity than the wild-type (24), suggesting a regulatory function for these residues. In the group 3, the C-term part comprises also a short helix of unknown function.

#### Contacts with REP

The REP_Ec_ GTAG motif, which is exclusively selected in SELEX and which tolerates no substitution for binding and catalytic activity, is heavily contacted by TnpA_Ec_ protein residues (group 2). These residues are distributed in the regions comprising β1 and surrounding β4 and also the C-terminal extremity (24) (Figure 6B). While these residues are well conserved in group 2, only some (Q95, D100 and R104) are relatively conserved in the group 3. In particular, G160 and E161, situated in the TnpA_Ec_ C-term tail and absent in the group 3, contact the last two bases of the GTAG motif. These differences may partly explain the discrepancy in GTAG requirement in the two groups.

In REP_Ec_ (group 2), the conserved mismatches forming a bulge in the middle of the stem A_12_A_13_-G_26_C_27_ were also important since mutations recreating perfect palindrome affected activity, the C_27_ is specifically contacted by the residue K82 situated in the conserved DNA binding α3 helix (Figure 6B, (24)). Nevertheless, these positions were not or only moderately selected by SELEX suggesting that different combinations of nucleotides are possible. And in the case of C_27_, we obtained a mixture C/T suggesting that a pyrimidine might be required at this position. Concerning group 3 REPs, exclusive selection of unique loop positions G_13_ (Sm), and T_13_ (Mb) and impact of mutations on activity demonstrated their crucial role (Figures 4 and 5). Since no structural data are available, we can only speculate relative to contacts with cognate TnpA_REP_. In spite of discrepancy, some parallel might be made between loop positions in small REPs of group 3 and unpaired positions in group 2 REP and residues on the equivalent DNA binding helix α3 might be responsible for these contacts. The same helix and downstream region might mediate cognate TnpA_REP_ binding to the group 3 REP stems as observed for TnpA_Ec._

### Binding to folded ssDNA hairpin

TnpA_REP_, as TnpA_IS_*_200_*_/IS_*_605_*, recognize their ss DNA REP substrates in a strand-specific manner. Only REP with characteristic features is bound and processed, iREP is not. In the group 3 REP, the conserved motif GTAG is clearly involved in strand discrimination, while its role in group 2 is more limited. Furthermore, the effect of single stranded features (loop or irregular zone as mismatches, bulge) is undeniable.

These properties echo those displayed by some proteins encoded by mobile genetic elements working on ss folded DNA such as Integrases IntI encoded by Integron, plasmid Relaxases and ss DNA Transposases TnpA_IS_*_200_*_/IS_*_605_*. For conjugative transfer, the Relaxase recognizes *oriT* as a single stranded folded hairpin (27). While contacts with stem remain non-specific, it establishes specific contacts with ss DNA cleavage region downstream of the hairpin. In the recombination reaction between the integron *attC* and *attI* sites, the ds DNA site *attC* is a ss folded structure from the bottom strand, reconstituting ds recombination site (28). In the crystal structure, Int establishes specific contacts with several flipped out bases in the *attC* site and these interactions are primordial for recombination (29). Moreover, efficient insertion of integron cassette is also influenced by two other unpaired regions of *attC* recombination sites (30). Recently, the impact of these structural specificity determinants of integron cassette has been refined using synthetic biology combined with large scale mutagenesis, next-generation sequencing and machine learning (31). This powerful approach will be a valuable tool to reexamine and to get a global view of specificity determinants and synthetic evolution pathways in diverse systems including REPtrons.

TnpA_REP_ are so far the closest relatives of TnpA_IS_*_200_*_/IS_*_605_*, among which transposases of IS*608* and IS*Dra2* are the most studied. To recognize the REP correct structure, TnpA_REP_ proteins contact loop or irregularities in the palindrome stem, as do ss transposases. IS*608* unpaired base T_17_ is sandwiched between aromatic residues in a hydrophobic pocket, whereas the T_10_ in the loop is specifically contacted by two residues (17). Similarly, IS*Dra2* transposase displayed contacts with T_14_ in the loop and a mismatched base within the stem (18). Thus, TnpA_REP_ proteins employ alternatively these binding determinants in combination with the conserved tetranucleotide GTAG. The last feature clearly distinguishes TnpA_REP_ from ss transposases that mostly contact exclusively the palindromes.

### Cleavage sites selection

Left and right cleavage sites of the IS*200*/IS*605* family members are selected via a network of peculiar complementary interactions with corresponding ‘guide’ sequences, which are tetranucleotides 5′ to the palindromes (19) (Supplementary Figure S1). Consequently, IS*608* cleavage sites could be modified by changing the corresponding ‘guide’ sequences, resulting also in retargeting of the IS (26). The position of the GTAG tetranucleotide in REPs could be equivalent to the ‘guide’ sequences. According to the proposed model of cleavage site selection based on examples of IS*608* and IS*Dra2*, the common CT and the Mb CA cleavage sites would be chosen via interactions with subsets of the conserved GTAG. TnpA_Mb_ turned out to be more flexible and cleaves REP substrate at both CT and CA sites. Thanks to this flexibility, we could explore this question and manage to vary REP_Mb_ CT and CA cleavage sites by changing certain positions in the GTAG motif. Although in these experiments the cleavage sites could be changed by that simple way, cleavage efficiency varies and it is not excluded that other factors would be involved.

In the cases of REPtrons Ec and Sm, similar attempts to change cleavage sites did not succeed (not shown). The REPtron Ec is known not to tolerate any GTAG mutation. In the case of Sm, while GTAG mutants still form complexes with TnpA_Sm_, they severely reduced cleavage, in particular when mutations concern the last two positions, consistent with their postulated role in cleavage site selection. We suppose that the CT site is indeed selected by the GTAG motif but that, in the case of the REPtron Ec, the GTAG is ‘protected’ from mutation by specific contacts with the protein, as shown by the structure. Alternatively, TnpA_Ec_ or TnpA_Sm_ could also accommodate CT dinucleotide into the catalytic site. This information was missing in the available structure.

### REPtrons and potential evolutionary route

REPtrons and IS*200*/IS*605* family members share major features. They exhibit an equivalent genetic structure in which coding sequences are bordered by palindromes, and encode proteins with a similar catalytic domain. Large scale phylogenetic analyses have confirmed the evolutionary relationship between TnpA_REP_ and TnpA_IS_*_200_*_/IS_*_605_* (13,20). TnpA_REP_ have been proposed to originate from ancient TnpA_IS_*_200_*_/IS_*_605_* ancestors in Enterobacteria and Pseudomonas where *tnpA*_REP_ are the most widespread. Alternatively, this distribution may reflect their successful establishment following arrival via horizontal transfer in these bacterial groups (ISfinder https://isfinder.biotoul.fr/), (32).

Our results here suggest that these two TnpA_REP_ groups co-evolve with their respective REP sequences. On the other hand, this does not seem to be the case with the IS*200*/IS*605* family, which includes two subgroups, one carries palindromes with irregularities (e.g. IS*608* and IS*Dra2*) whereas another one is associated with perfect palindromic ends (e.g. IS*200*, IS*1451*). Yet TnpA_IS_*_200_*_/IS_*_605_* appear very homogenous, no distinction being observable in corresponding transposases sequences (ISfinder). It will be interesting to know whether the two described REPtrons groups here have evolved from a common ancestor, common with IS*200*/IS*605* family members or not.

In spite of the close relationship between TnpA_REP_ and TnpA_IS_*_200_*_/IS_*_605_*, while *tnpA*_IS_*_200_*_/IS_*_605_* exhibit typical behavior of IS transposase genes, *tnpA*_REP_ are, in many respects, very close to housekeeping genes (13), supporting the previous consideration of TnpA_REP_ as the first described bacterial domesticated transposases. The maintenance of *tnpA*_REP_ in bacterial genomes also implies that they have been coopted to fulfil functions benefic to the host cell. Diverse documented functions of REP sequences in cell physiology suggest their roles in improving fitness of bacterial host in a given niche or environment. This notion has been reinforced by a recent genome-wide CRISPRi analysis in *E. coli* using the catalytic null mutant of the Cas9 RNA-guided nuclease (CRISPR-dCas9) for silencing genes of interest (33). Interestingly, this study has revealed fitness defect caused by dCas9 binding at different REP sequences. Works are in progress to decipher the dissemination pathway of these important elements.

## Supplementary Material

gkab524_Supplemental_File
