# Supplementary material for: TnpAREP and REP sequences dissemination in bacterial genomes: REP recognition determinants
Source: Nucleic Acids Res. 2021 Jun 23;49(12):6982–95. doi: 10.1093/nar/gkab524 (PMC8266576; doi:10.1093/nar/gkab524)
Supplement: gkab524_Supplemental_File [file gkab524_supplemental_file.pdf]

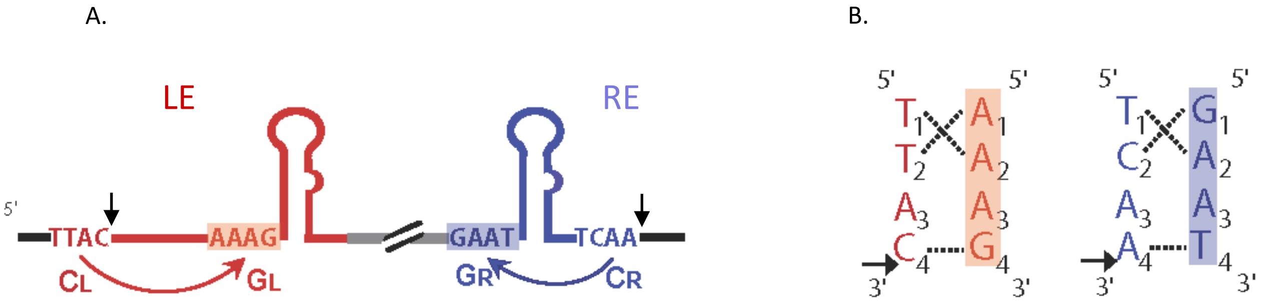

Figure S1: IS*608* cleavage site selection model, adapted from (Guynet et al., 2009).

A. Left end (LE) and right end (RE) are represented in red and blue, respectively. Left and right cleavage sites C_L and C_R are indicated; “guide” sequences G_L and G_R are shown on a red and blue background, respectively. Black arrows indicate cleavage positions. **B**. Left and right IS end cleavage sites C_L and C_R are selected by interactions with corresponding “guide” sequences G_L and G_R, respectively, via a network of complementarity. Black arrows indicate cleavage positions.

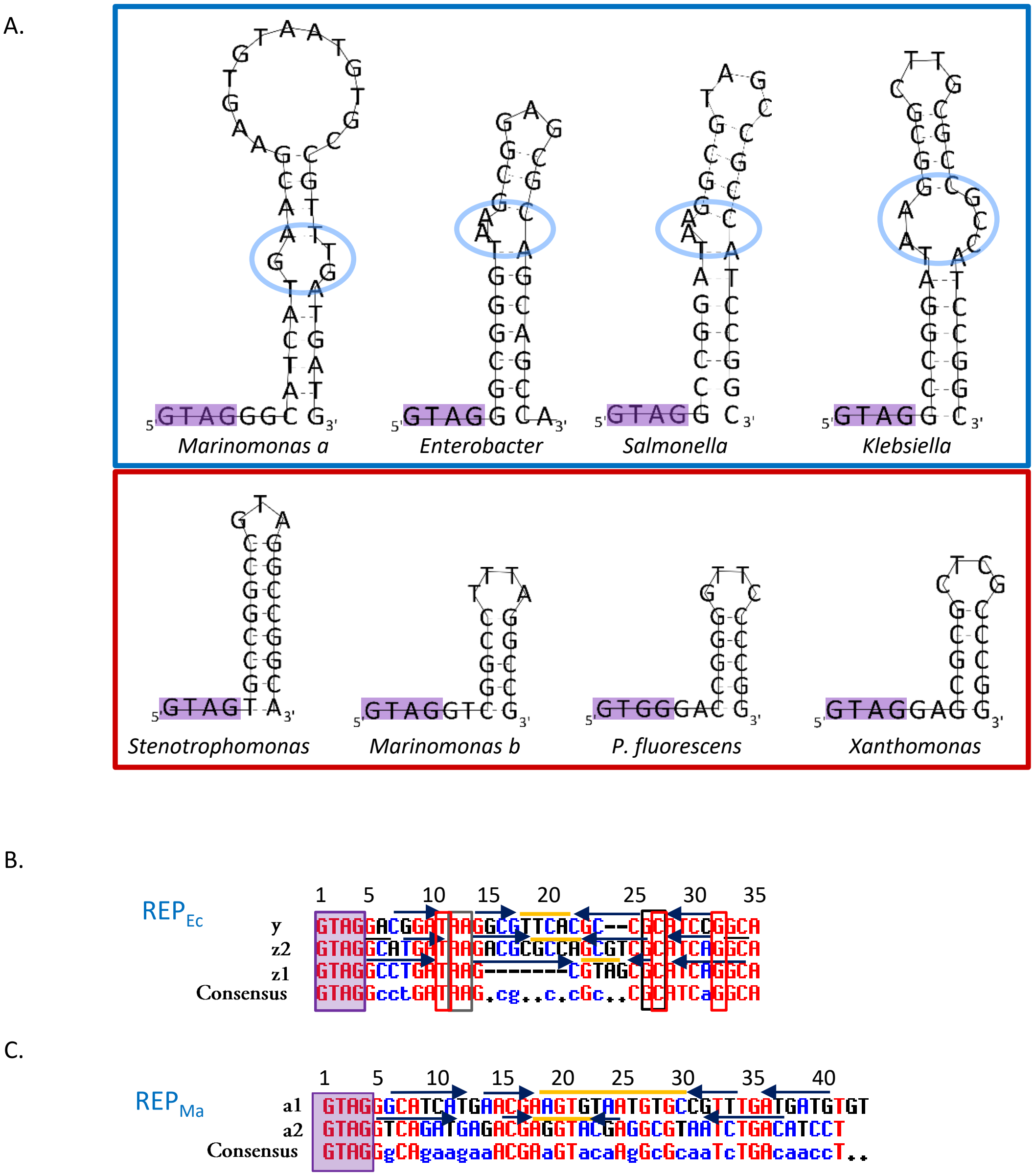

Figure S2: REPs in groups 2 and 3

A. REPs of the group 2 (boxed in blue) and of the group 3 (boxed in red). Irregularities on the group 2 REP stem are circled in blue. **B**. *Escherichia coli* MG1655 Ec REPtron, sequences of y, z1 and z2 REP and alignment of their consensus. Conserved positions equivalent to y T_{11}, C_{27} and G_{32} contacted by $TnpA_{Ec}$ are boxed in red. Loop sequences are marked in orange. Conserved mismatches forming a bulge are boxed in black. **C**. Alignment of REP_{Ma1} and REP_{Ma2} from group 2 *Marinomonas* sp. MWLY1 REPtron Ma.

Figure S3

A.

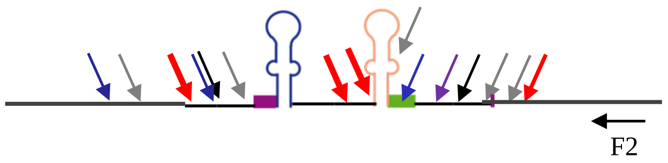

CCAGGTATTCAGGTCAATCCGGGTAAAATTCATGGTTATTCCTTCTGATTAATAGTGAAA
AATATTAATAATCAGAAGGCAGTCTGGTTGTCTCAATGGGTAACATTCCGTCCTCCGTAA
GCTGTTTGGTATTCAGTAATAATACCCTATACGGGCTTAATCTGTATTAAGCCCGGCTTT
ATTTATTCCGGCCAATCATCCGCAAACACATAGCGGATCAGTT**CT**GCGATATTCAGACAT
GGCGACTCCTTTTTCTCAACGGTAAGAAGAAAAGGAAAGGTTGGCAATGAGGTTGTTTTA
TATCGGGAAAGCGGCTCGCAAATATTCGCGGGCTC**GTAG**GCATGATAAGACGCGCCAGCG
TCGCATCAGGCAC**CT**GCGTACAA**CT**GCCGGATGCGGCGTAAACGCCTTATCCGTC**CTAC**A
AAATCGTCTCTTCCGTCAAAATCCTGCGCGCGCCCAAAAAATGGTCCTGCCAGAAAGGTT
CGGCTAATCGGCTTATCCGGGCTGCAGGAATTCGATTTGCGCTAGTGCAAAAATTACCAA
ATTTGCCTAATGACTTTAATAAAATCAAGCTTATCGATACCGTCGAC**CT**CGAGGGGGGGC
CCGGTAC**CCAATTCGCCCTATAGTGAG**

B.

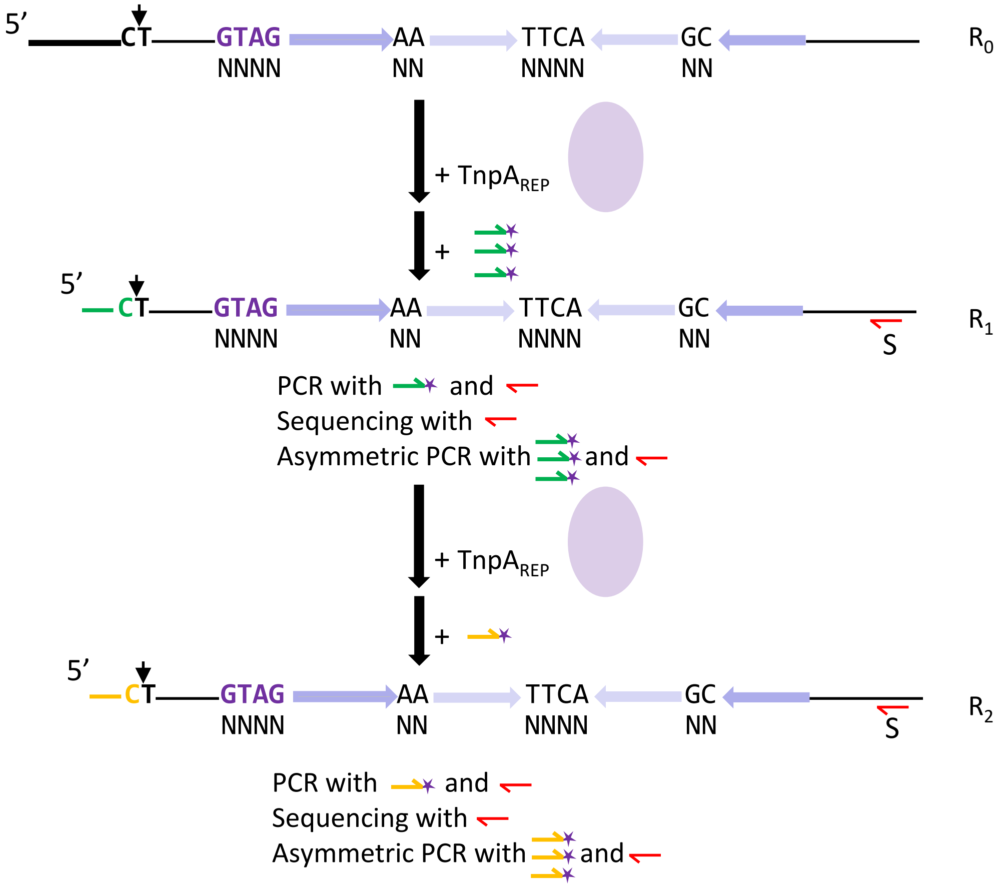

Figure S3: CST and CST-based SELEX

A. CST-based mapping of cleavage sites on ss phagemid substrate. Top: cartoon of REPtron Ec 5′ BIME structure and mapped cleavage sites. Bottom: BIME sequence and adjacent region where position of substrate-specific primer F2 is in bold, REP and IREP sequences are in blue and orange, respectively, GTAG and CTAC motifs are in bold violet and green (CT in the CTAC motif is also cleaved). Mapped cleavage sites are in red, major sites in bold.

B. CST-based SELEX. Linear presentation of different oligonucleotide substrates (R_0) carrying **an unique 5′ cleavage site** (CT) and REP sequence (example based on REP_{Ec} structure where bold blue arrows represent complementary sequences constituting REP stem, interrupted by a bulge) with different distinct degenerate motifs (Nn). Substrates are incubated with $TnpA_{REP}$ and treated as described for CST. After the first PCR amplification, selected substrates were sequenced with a common substrate-specific primer S (first round, R_1). Ss substrates for the next round were prepared by amplification from the resulting products (R_1) see Materials and Methods. For each round, different “attacking” oligonucleotides were used, all carrying a 3′C permitting reconstitution of the cleavage site for the next round.

R$_0$ *TGCGATTTT***N$_1$N$_2$N$_3$N$_4$***GACGGA*

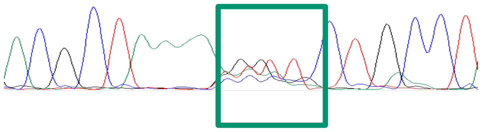

R$_1$ *TGCGATTTT***G$_1$T$_2$A$_3$G$_4$***GACGGA*

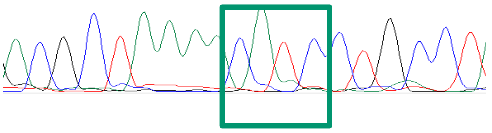

1.

Raw H_f	G	C	T	A
	71.518616	47.58716	60.330906	85.10275
	99.01517	78.83921	66.16769	61.552097
	83.467926		58.29558	92.40365
	87.04616		62.13774	
	92.23951		66.568184	

Raw H_f	G	C	T	A
	57.940075	62.322495	60.355106	66.05037
	91.411964	89.099754	93.388756	49.275555
	68.98452		82.489136	71.996414
	60.945698		72.42838	
	78.101074		64.9068	

Raw H_d	G	C	T	A
N1	11.868318	19.324127	19.990467	17.499918
N2	14.500667	35.337124	25.09317	22.171158
N3	13.151994	33.978878	20.273228	33.850903
N4	12.35254	14.355808	14.995016	34.52713

Raw H_d	G	C	T	A
N1	63.31935	0.729149	1.3034401	1.866446
N2	7.8927402	1.1680177	101.976166	0.5247616
N3	0.645137	2.7178514	10.306519	59.604015
N4	64.30367	3.6632998	0.26269427	2.54105

2.

H_r	86.6574764	78.83921	62.70002	88.7532
H_e				
N1	13.6956654	24.5108075	31.8827123	19.7175065
N2	16.7333133	44.8217632	40.020992	24.9806858
N3	15.1769871	43.098958	32.3336867	38.1404873
N4	14.2544423	18.2089699	23.9154884	38.9024058

H_r	71.4766662	75.7111245	74.7136356	69.023392
H_e				
N1	88.5874417	0.96306719	1.74458128	2.70407748
N2	11.0424011	1.5427293	136.489364	0.76026632
N3	0.90258407	3.58976494	13.7946961	100.841197
N4	89.9645624	4.83852251	0.35160151	3.6814331

3.

F_0				
N1	**0.15250161**	0.27292852	0.35501488	0.21955498
N2	0.13221984	0.35416334	**0.3162296**	0.19738722
N3	0.1178794	0.33474888	0.2511352	**0.29623652**
N4	**0.14960377**	0.19110748	0.25099875	0.40829001

F_1				
N1	0.94242794	0.01024549	0.01855954	0.02876704
N2	0.07369719	0.0102962	0.91093257	0.00507403
N3	0.00757658	0.03013362	0.11579703	0.84649278
N4	0.91023973	0.048955	0.00355742	0.03724785

4.

$E_{1,0}$	G	C	T	A
N1	6.17978995	0.03753908	0.0522782	0.13102429
N2	0.55738378	0.02907191	2.880605	0.02570598
N3	0.06427395	0.09001858	0.46109437	2.85748961
N4	6.08433695	0.25616477	0.01417306	0.09122891

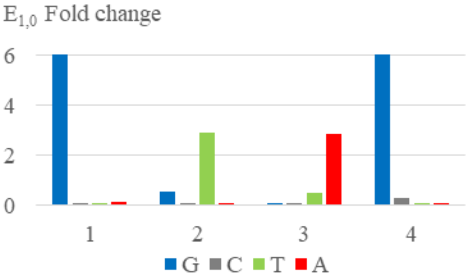

5.

Position	Score (Variance)	Level
N1	8.35842531	+++
N2	2.41212618	+++
N3	1.83968434	+++
N4	7.52000379	+++

6.

EcGTAG Fig3A		Ecbulge Fig3B		Ecloop Fig3C		SmGTAG Fig4D		Smloop Fig4E		MbGTAG-CT Fig5B		MbGTAG-CA Fig5B		Mbloop Fig5F	
G1	+++	T12	+	T18	-	G1	+++	G13	+++	G1	-	G1	++	T12	++
T2	+++	A13	+	T19	-	T2	++	C14	+	T2	-	T2	++	T13	+++
A3	+++	G26	-	T20	-	A3	++	T15	-	A3	+++	A3	++	T14	-
G1	+++	CT27	+	A21	-	G1	++			G1	+++	G1	+++	A15	+++

Figure S3C

Fig S3C. Example of REP_{Ec} GTAG SELEX and Scores of other experiments

1. SELEX on REP_{Ec} GTAG motif. Sanger sequencing profiles of initial R_0 and first round R_1 selected substrates where the REP_{Ec} GTAG motif sub-window is boxed in green (top left and right). Extraction of raw heights and calculation of reference heights $\mathbf{H_r}$ of fixed positions, respectively (bottom).

2. Extraction of raw heights of degenerate positions and calculation of external normalized heights $\mathbf{H_e}$

3. Calculation of internal normalized fractions $\mathbf{F_0}$ and $\mathbf{F_1}$ of degenerate motifs in the initial and the final chromatograms.

4. Calculation of Enrichment factor (Fold change) $\mathbf{E_{1,0}}$ and Plot. Lower $E_{1,0}$ of T_2 and A_3 are due to higher F_0 (see 3, in bold) for these bases.

5. Calculation of **Scores** of Selex as Variance of $E_{1,0}$ at each position and levels of selection.

6. Levels of selection for all SELEX analyses in this study.

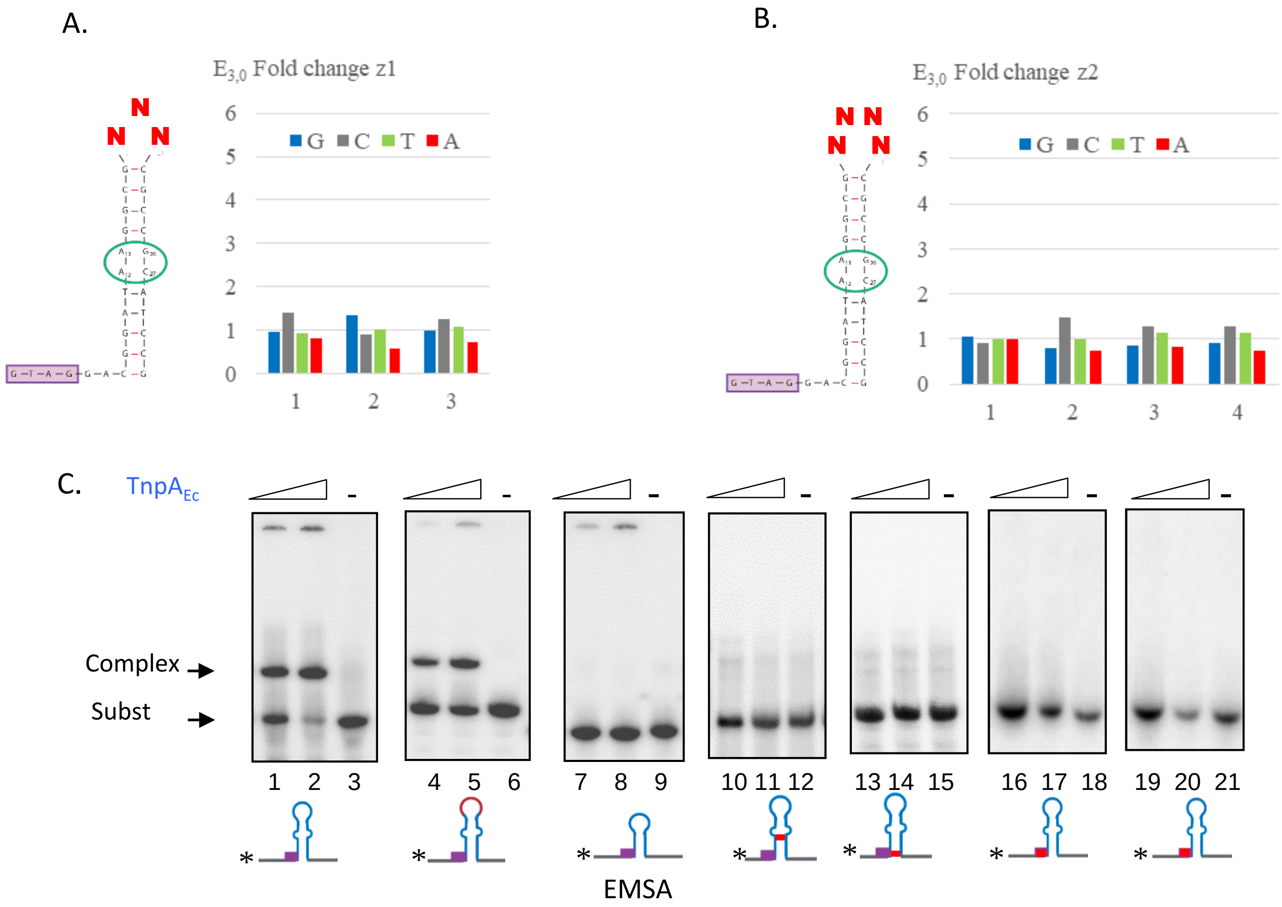

Figure S4: CST-based SELEX on REP_{Ec}

A. CST-based SELEX on REP_{Ec} z1 loop: enrichment with low scores at R3.

Z1loop selex

ACACG**CT**GCGATTTT**GTAG**GTAGGCCTGATAAGCNNNGCGCATCAGGCATAATTAACGT
GATCATTCACAT*GTTGCGACGACGAAGCGATT*TT

B. CST-based SELEX on REP_{Ec} z2 loop: enrichment with low scores at R3.

Z2loop selex

ACACG**CT**GCGATTTT**GTAG**GCATGATAAGACGCNNNNGCGTCGCATCAGGCA
TAATTAACGTGATCATTCACAT*GTTGCGACGACGAAGCGATT*TT

C. EMSA experiment performed with increasing $TnpA_{Ec}$ concentrations (2 and 4 µM) indicated by the triangle or in its absence (-) on wt y REP_{Ec} substrate (lanes 1-3), substrate carrying mutated loop (lanes 4-6), substrate deleted for superior half (lanes 7-9), substrates carrying reverse sequence of conserved G32 (lanes 10-12) and of T11 (lanes 13-15), substrates carrying individual mutations in the GTAG motif, GTGG (lanes 16-18) and GTAC (lanes 19-21) respectively . Bottom: cartoon representing REP structures in blue with mutations in red.

•B268i (61) wt

GTAGGACGGATAAGGCGTTTACGCCGCATCCGGCAGTTGTACGCAGGTGCCTGATGCGACG

•B2689 mut loop

GTAGGACGGATAAGGCGTAAACGCCGCATCCGGCAGTTGTACGCAGGTGCC**CT**GATGCGACG

•B2688 (53) del upper half

•**GTAG**GACGGATAAGCATCCGGCAGTTGTACGCAGGTGCC**CT**GATGCGACG

•E268 (mut conserved G32)

GTAGGA**G**GGATAAGGCGTTTACGCCGCATCC**C**GCAGTTGTACGCAGGTGCC**CT**GATGCGACG

•F268 (mut conserved T11)

GTAGGACGGA**A**AAGGCGTTTACGCCGC**T**TCCGGCAGTTGTACGCAGGTGCC**CT**GATGCGACG

•B268i (61) GTGG

GTGGGACGGATAAGGCGTTTACGCCGCATCCGGCAGTTGTACGCAGGTGCCTGATGCGACG

•B268i (61) GTAC

GTACGACGGATAAGGCGTTTACGCCGCATCCGGCAGTTGTACGCAGGTGCCTGATGCGACG

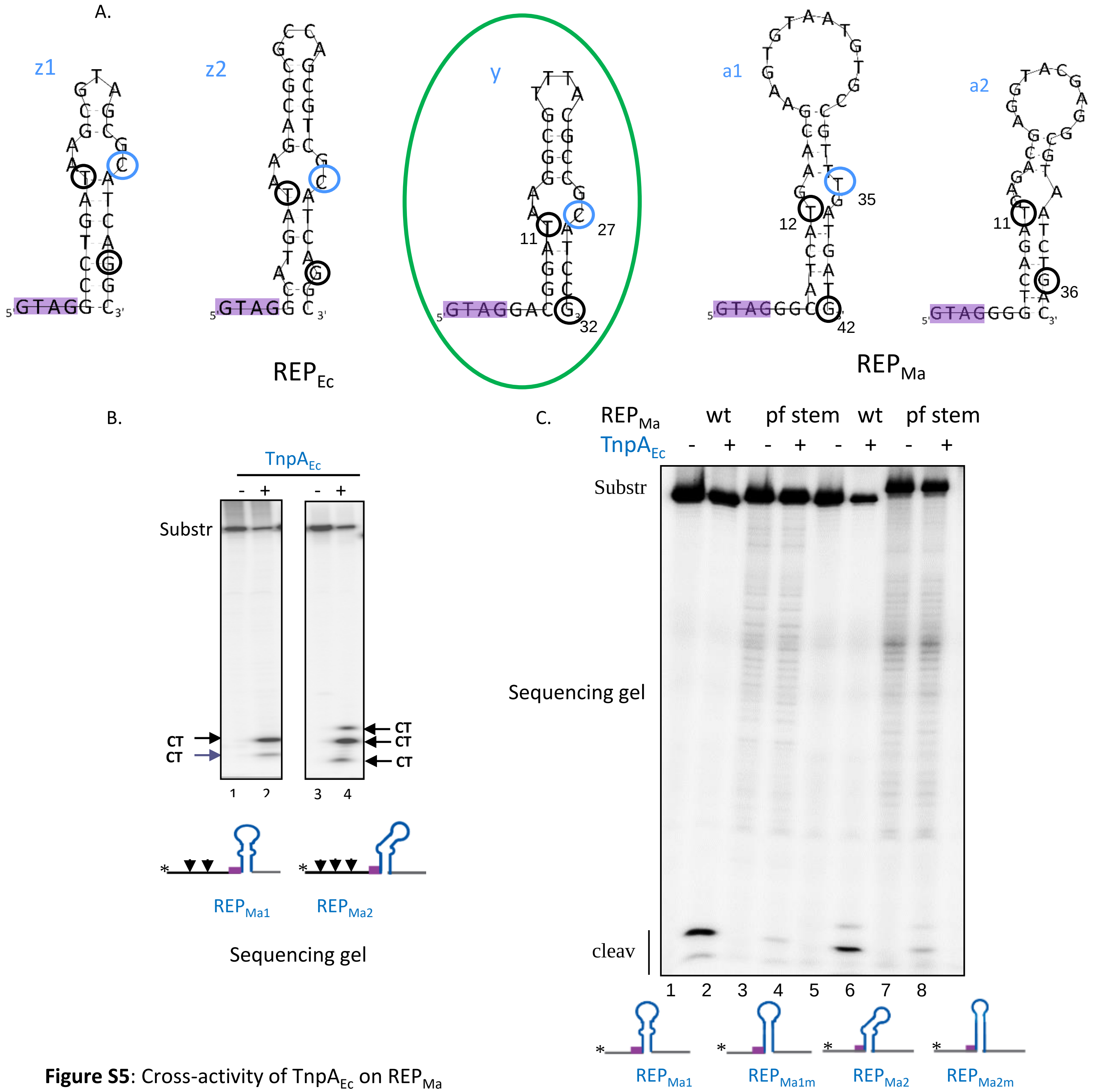

Figure S5: Cross-activity of TnpA$_{Ec}$ on REP$_{Ma}$

A. Potential common features concern T$_{12}$ (REP$_{Ma1}$) and T$_{11}$ (REP$_{Ma2}$) and G$_{42}$ (REP$_{Ma1}$), G$_{36}$ (REP$_{Ma2}$) equivalent of T$_{11}$ and G$_{32}$ in y REP$_{Ec}$ (circled in black), respectively. Relative to the base C$_{27}$ (y REP$_{Ec}$, circled in blue), pyrimidine base T$_{35}$ (REP$_{Ma1}$) as shown by y REP$_{Ec}$ SELEX in the bulge region.

B. Cleavage of 60 nts REP$_{Ma1}$ and 61 nts REP$_{Ma2}$ by TnpA$_{Ec}$ (lanes 2 and 4 respectively).

C. Abolishment of the bulged region affected TnpA$_{Ec}$ activity on REP$_{Ma1}$ and REP$_{Ma2}$. TnpA$_{Ec}$ mediated cleavage on wt REP$_{Ma1}$ (lane 2) and wt REP$_{Ma2}$ (lane 6) compared to mutant substrates carrying a perfect stem REP$_{Ma1m}$ (lane 4) and REP$_{Ma2m}$ (lane 8).

•MA2 (60) wtREPMa1

GTCAT**CTTCT**TTTTC**GTAG**GGCATCATGAACGAAGTGTAATGTGCCGTTTGATGATGTGT

•MA20 REP$_{Ma1m}$

GTCAT**CTTCT**TTTTC**GTAG**GGCATCAT**CA**AACGAAGTGTAATGTGCCGTTTGATGATGTG

•MA5(61) wtREPMa2

CAACC**CTGCTCT**TTTCCC**GTAG**GTCAGATGAGACGAGGTACGAGGCGTAATCTGACATCCT

•MA50 REP$_{Ma2m}$

CAACC**CTGCTCT**TTTCCC**GTAG**GTCAGATGAGACGAGGTACGAGGCGT**CTC**ATCTGACATCCT

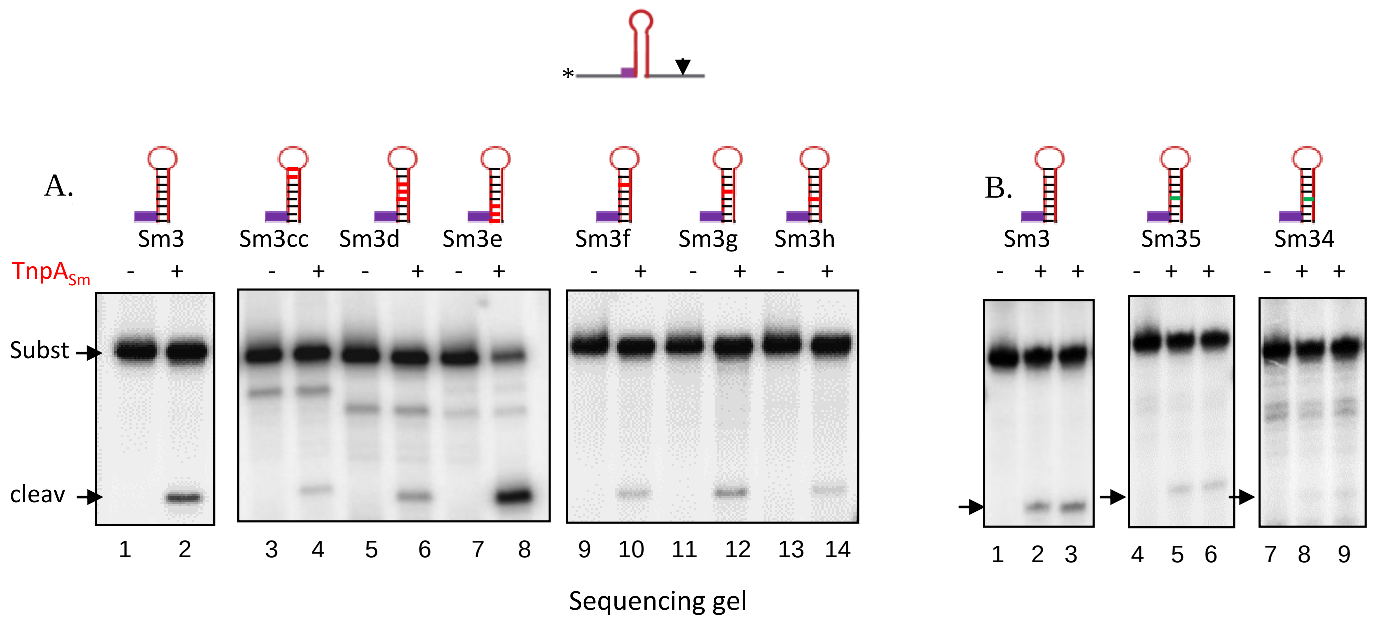

Figure S6: Importance of the REP$_{Sm}$ stem

A. Cleavage of TnpA$_{Sm}$ on wt 55 nts REP$_{Sm}$ substrate (lanes 1-2), substrate carrying stem with inversion of 2 superior base pairs (lanes 3-4), inversion of next 3 base pairs (lanes 5-6), inversion of bottom 3 base pairs (lanes 7-8) and inversion of individual 3rd, 4th and 5th positions (lanes 9-10, 11-12, 13-14) respectively**.** Top: Cartoons representing REP structures with inverted or mismatched positions in red and green, respectively.

B. Sm3 (55nt)

AGGTCCG**GTAG**TGCCGGCCGCTGGCCGGCAACCTCATTCAA**CT**TCAGCAACCCAC

Sm3cc complement of top 2 bases

AGGTCCG**GTAG**TGCCGGGGGCTCCCCGGCAACCTCATTCAA**CT**TCAGCAACCCAC

Sm3d (55nt) complement of 3 bases in the middle of stem

AGGTCCG**GTAG**TGCGCCCCGCTGGGGCGCAACCTCATTCAA**CT**TCAGCAACCCAC

Sm3e (55nt) complement of the 3 bases at the bottom

AGGTCCG**GTAG**ACGCGGCCGCTGGCCGCGTACCTCATTCAA**CT**TCAGCAACCCAC

Sm3f complement of the 3rd base

AGGTCCG**GTAG**TGCCGCCCGCTGGGCGGCAACCTCATTCAA**CT**TCAGCAACCCAC

Sm3g complement of the 4th base

AGGTCCG**GTAG**TGCCCGCCGCTGGCGGGCAACCTCATTCAA**CT**TCAGCAACCCAC

Sm3h complement of the 5th base

AGGTCCG**GTAG**TGCGGGCCGCTGGCCCGCAACCTCATTCAA**CT**TCAGCAACCCAC

B. Effect of mismatches in the stem of REP$_{Sm}$ on cleavage. Cleavage reactions carried with wt substrate (lanes 1-3), substrate carrying mismatch at 5th position G-T (lanes 4-6) and mismatch at 5th position C-A (lanes 7-9) respectively.

Sm35 (55nt) mismatch 5th G-T

AGGTCCG**GTAG**TGCCGGCCGCTGGCCTGCAACCTCATTCAA**CT**TCAGCAACCCAC

Sm34 (55nt) mismatch 5th C-A

AGGTCCG**GTAG**TGCAGGCCGCTGGCCGGCAACCTCATTCAA**CT**TCAGCAACCCAC

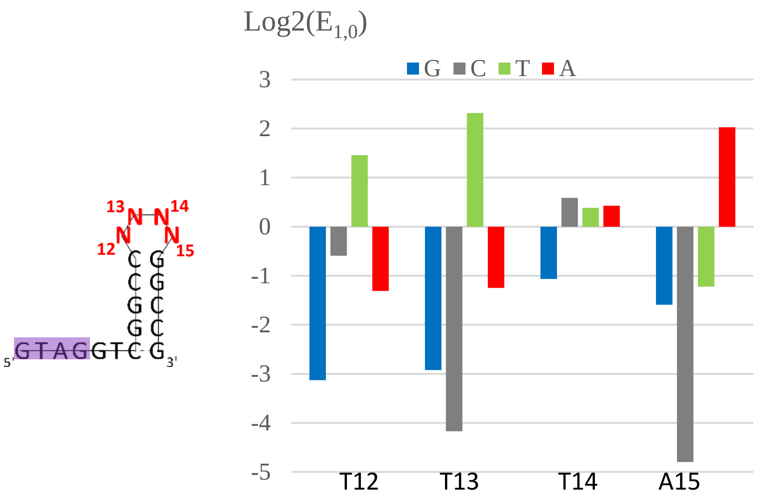

Fig. S7: SELEX of REP_{Mb} loop sequence

Exclusion of the REP_{Mb} loop sequence at R1 selection, where $E_{1,0}$ is enrichment factor, G, C, T, A are in blue, grey, green and red respectively. The same schema as described in Fig. 5F with $N_{12}N_{13}N_{14}N_{15}$ degenerated loop sequence. Underneath: initial sequence of the motif.

MnselCT2

TGATTTG**CT**TTTT**GTAG**GTCGGCCNNNNGGCCGTCAATCGATCGAATTAACGT
GATGATTGAGAT*GTTGCGACGACGAAGCGATT*

MnselCA

TGTGATT**CA**TTTT**GTAG**GTCGGCCNNNNGGCCGTCAATCGATCGAATTAACGT
GATGATTGAGAT*GTTGCGACGACGAAGCGATT*

$TnpA_{REP}$ and REP sequences dissemination in bacterial genomes:

REP recognition determinants

Supplementary materials

CST and CST-based SELEX: SELEX efficiency estimation

This procedure serves **to estimate** the level of enrichment or exclusion of each base at each position in degenerate motifs. Results are based on the heights of peaks from a window of Sanger bulk sequence. Taking into account the variability in peak heights between nucleotides in fixed positions and the inter-dependence of nucleotide content at the same position in degenerate motif requires external (i.e. outside the degenerate motif) and internal (i.e. at each position within the degenarate motif) normalisation. For this purpose:

1. Extraction of peak height: From a window of Sanger bulk sequence carrying the degenerate motif with surrounding sequences, heights of all peaks are extracted using Plot digitizer. These values are considered as raw heights $\mathbf{H_f}$ and $\mathbf{H_d}$ of fixed and degenerate positions, respectively. Peak heights are extracted from the sequencing data of the initial substrate R_0 and that obtained after N rounds of SELEX procedure R_N.

2. External normalisation: This step serves to calculate the height of a given degenerate base compared to the maximal (of fixed positions) height. A reference height $\mathbf{H_r}$ for each base G,C,T,A is calculated mainly as the mean of peak heights from a subset of fixed positions where the nucleotide is present $\mathbf{H_r=MEAN(H_f)}$. The normalised height at each degenerate position is calculated for each base $\mathbf{H_e=100*H_d/H_r}$ where $\mathbf{H_d}$ and $\mathbf{H_r}$ are the raw peak height of each nucleotide at a given position and the reference peak height for this nucleotide**.** This normalisation external to the degenerated region accounts for the variability in peak heights between nucleotides in the fixed positions.

3. Internal normalisation: $\mathbf{H_e}$ is further normalised inside each position (internal normalisation) to calculate the fraction of each base at a given position $\mathbf{F=H_e/SUM(H_e)}$ where H_e is external normalised height for each base at the same position.

4. Enrichment level: Enrichment level of each base at each position can be expressed as the Enrichment factor (Fold change) $\mathbf{E_{N,0}=F_N/F_0}$ where F_N and F_0 are internal normalised fractions of each base, at a given position in the final (N) and initial (0) chromatograms**.** Here, we consider the mean of $E_{N,0}$ of several independent experiments.

5. Score calculation: To evaluate the level of selection at each position of the degenerate motif, we consider the score of each position as $\mathbf{S=VAR.S(E_{N,0})}$ where $E_{N,0}$ concerns all bases at the same position. We arbitrarily classify these values into 4 categories as follows:

**Variance**	>1.5	[1.5;0.5)	[0,5;0.2]	<0.2
**Score**	+++<br>High	++<br>Good	+<br>Medium	-<br>Low

List of substrates

Oligonucleotides substrates (principal figures)
Ec
Fig 3A-B
268sel4 selexGTAG
ACACGC**CT**GCGATTTT**NNNN**GACGGATAAGGCGTTTACGCCGCATCCGGCATAATTAACGT
GATCATTCACAT*GTTGCGACGACGAAGCGATT*TT
268sel3 selexbulge
ACACGC**CT**GCGATTTT**GTAG**GACGGAT**NN**GGCGTTTACGCC**NN**ATCCGGCATAATTAACGTGATCATTCACATTTA
ACGTGATCATTCACAT*GTTGCGACGACGAAGCGATT*TT
Fig 3C
B268i (61) wt
GTAGGACGGATAAGGCGTTTACGCCGCATCCGGCAGTTGTACGCAGGTGCCTGATGCGACG
C268 (61) mutbulge
GTAGGACGGATTAGGCGTTTACGCCGTATCCGGCAGTTGTACGCAGGTGCCTGATGCGACG
D268 (61) mutbulge
GTAGGACGGATATGGCGTTTACGCCTCATCCGGCAGTTGTACGCAGGTGCCTGATGCGACG
Fig 3D
268sel selexyloop
ACACGC**CT**GCGATTTT**GTAG**GACGGATAAGGCG**NNNN**CGCCGCATCCGGCATAATTAACGT
GATCATTCACAT*GTTGCGACGACGAAGCGATTTT*
Fig 3E
B268i (61) wt
GTAGGACGGATAAGGCGTTTACGCCGCATCCGGCAGTTGTACGCAGGTGCCTGATGCGACG
B2689 mutloop
GTAGGACGGATAAGGCGTAAACGCCGCATCCGGCAGTTGTACGCAGGTGC**CT**GATGCGACG
B2680 mutupperstem
GTAGGACGGATAACCGCTAAAGCGGGCATCCGGCAGTTGTACGCAGGTGC**CT**GATGCGACG
B2688 (53) delupperstemandloop
GTAGGACGGATAAGCATCCGGCAGTTGTACGCAGGTGC**CT**GATGCGACG
E268 (mut conserved G32)
GTAGGA**G**GGATAAGGCGTTTACGCCGCATCC**C**GCAGTTGTACGCAGGTGC**CT**GATGCGACG
F268 (mut conserved T11)
GTAGGACGGA**A**AAGGCGTTTACGCCGC**T**TCCGGCAGTTGTACGCAGGTGC**CT**GATGCGACG

Sm
Fig 4A
Sm3 (55nt) wt
AGGTCCG**GTAG**TGCCGGCCGCTGGCCGGCAACCTCATTCAA**CT**TCAGCAACCCAC
Sm3c
AGGTCCG**GTAG**TGCCGGCCGCTGGCCGGCAACCTCATTCAATTTCAGCAACCCAC
bSm3 (55nt)
AGGTCCG**GTAG**TGCCGGCCATCGGCCGGCAACCTCATTCAA**CT**TCAGCAACCCAC
Fig 4B-C

Sm3 (55nt) wt
AGGTCCG**GTAG**TGCCGGCCGCTGGCCGGCAACCTCATTCAA**CT**TCAGCAACCCAC
Sm3CTAG (55nt)
AGGTCCG**CTAG**TGCCGGCCGCTGGCCGGCAACCTCATTCAA**CT**TCAGCAACCCAC
Sm3GCAG (55nt)
AGGTCCG**GCAG**TGCCGGCCGCTGGCCGGCAACCTCATTCAA**CT**TCAGCAACCCAC
Sm3GTTG (55nt)
AGGTCCG**GTTG**TGCCGGCCGCTGGCCGGCAACCTCATTCAA**CT**TCAGCAACCCAC
Sm3GTAC (55nt)
AGGTCCG**GTAC**TGCCGGCCGCTGGCCGGCAACCTCATTCAA**CT**TCAGCAACCCAC
Fig 4D
Smsel3 selexGTAG
ATTCAA**CT**TCACCG**NNNN**TGCCGGCCGCTGGCCGGCAACATCATTCCAATTAACGTGATCATTCACATAATTAAC
GTGA*GTTGCGACGACGAAGCGATT*
Fig 4E
Smsel2 selexloop
GATTCAA**CT**TCACCG**GTAG**TGCCGGCC**NNN**GGCCGGCAACATCATTCCAATTAACGTGATCATTCACATAATTAA
CGTGA*GTTGCGACGACGAAGCGATT*

Fig 4F
Sm3 (55nt) wt
AGGTCCG**GTAG**TGCCGGCCGCTGGCCGGCAACCTCATTCAA**CT**TCAGCAACCCAC
Sm3a (55nt) mutloop
AGGTCCG**GTAG**TGCCGGCCCCTGGCCGGCAACCTCATTCAA**CT**TCAGCAACCCAC

Mb
Fig 5A
Mn1 (52nt)wt
TGATT**CA**TG**CT**TTTT**GTAG**GTCGGCCTTTAGGCCGTCAAGAA**CT**CA**CA**TTTA
Mn1b (52nt)
TGATT**CA**TGTTTTTT**GTAG**GTCGGCCTTTAGGCCGTCAAGAATTAA**CA**TTTA
Fig 5B
MnselCT3 selexGTAGCT
TGATTTG**CT**TTTT**NNNN**GTCGGCCTTTAGGCCGTCAATCGATCGAATTAACGTGATGATTGAGAT*GTTGCGACGA*
CGAAGCGATT
MnselCA4 selexGTAGCA
TGATT**CA**TGCGTTTT**NNNN**GTCGGCCTTTAGGCCGTCAATCGATCGAATTAACGTGATGATTGAGAAATTAATAT
GAT*GTTGCGACGACGAAGCGATT*
Fig 5C
Mn4b (39nt) wt CAsubstrate
TT**GTAG**GTCGGCCTTTAGGCCGTCAAGAATTTA**CA**TTTA
M3(39nt)
TT**GTGG**GTCGGCCTTTAGGCCGTCAAGAATTTA**CA**TTTA
M4(39nt)
TT**GTGG**GTCGGCCTTTAGGCCGTCAAGAATTTA**CC**TTTA
M9 (39nt)
TT**GTAC**GTCGGCCTTTAGGCCGTCAAGAATTTA**GT**TTTA
M10 (39nt)
TT**GTAC**GTCGGCCTTTAGGCCGTCAAGAATTTA**GA**TTTA
M8 (39nt) no cleavage site
TT**GTAC**GTCGGCCTTTAGGCCGTCAAGAATTTACATTTA
Fig 5D
Mn1 (52nt)wt not shown
TGATT**CA**TG**CT**TTTT**GTAG**GTCGGCCTTTAGGCCGTCAAGAA**CT**CA**CA**TTTA
iMn1alix (52nt) not shown
TGATT**CA**TG**CT**TTTT**GTAG**GTGCCGGTTTACCGGCTCAAGAA**CT**CA**CA**TTTA
t1Mn1 (52nt) complement of 1st position not shown
TGATT**CA**TG**CT**TTTT**GTAG**GTCGGCGTTTACGCCGTCAAGAA**CT**CA**CA**TTTA
t2Mn1 (52nt) complement of 5th position not shown
TGATT**CA**TG**CT**TTTT**GTAG**GTGGGCCTTTAGGCCCTCAAGAA**CT**CA**CA**TTT

Mn4(39) wt
TT**GTAG**GTCGGCCTTTAGGCCGT**CA**AGAA**CTCACA**TTTA
Mn4dd complement of the central 3 bases not shown
TT**GTAG**GTCCCGCTTTAGCGGGT**CA**AGAA**CTCACA**TTTA
Mn4ff complement of 2nd base
TT**GTAG**GTCGGGCTTTAGCCCGT**CA**AGAA**CTCACA**TTTA
Mn4gg complement of 3rd
TT**GTAG**GTCGCCCTTTAGGGCGT**CA**AGAA**CTCACA**TTTA
Mn4hh complement of 4th
TT**GTAG**GTCCGCCTTTAGGCGGT**CA**AGAA**CTCACA**TTTA
Fig 5E
Mn4(39) wt
TT**GTAG**GTCGGCCTTTAGGCCGT**CA**AGAA**CTCACA**TTTA
Mn44(39/34+5)mismatch 4th position G-A
TT**GTAG**GTC**G**GCCTTTAGGC**A**GTCAAGAA**CTCACA**TTTA
Mn45(39/34+5)mismatch 4th position T-C
TT**GTAG**GTC**T**GCCTTTAGGC**C**GT**CA**AGAA**CTCACA**TTTA

Fig 5F
MnselCT2 selexloopCT
TGATTTG**CT**TTTT**GTAG**GTCGGCC**NNNN**GGCCGTCAATCGATCGAATTAACGTGATGATTGAGAT*GTTGCGACGA CGAAGCGATT*
MnselCA selexloopCA not shown
TGTGATT**CA**TTTT**GTAG**GTCGGCC**NNNN**GGCCGTCAATCGATCGAATTAACGTGATGATTGAGAT*GTTGCGACGA CGAAGCGATT*
Fig 5G
Mn4(39) wt
TT**GTAG**GTCGGCCTTTAGGCCGT**CA**AGAA**CTCACA**TTTA
Mn4bb mutloop excluded base
TT**GTAG**GTCGGCCGTTAGGCCGT**CA**AGAA**CTCACA**TTTA
Mn4aa mutloop excluded base
TT**GTAG**GTCGGCCTGTAGGCCGT**CA**AGAA**CTCACA**TTTA
Mn4ee mutloop excluded base
TT**GTAG**GTCGGCCTCTAGGCCGT**CA**AGAA**CTCACA**TTTA
Mn4cc mutloop neutral position
TT**GTAG**GTCGGCCTTNAGGCCGT**CA**AGAA**CTCACA**TTTA
Mn46 ATTTloop
TT**GTAG**GTCGGCCATTTGGCCGT**CA**AGAA**CT**CA**CA**TTTA

Oligonucleotides for CST and CST-based SELEX
Attacking (A)
457
Cagtgtcacaatggccttac
534
CAGTGTCACAATGCCACAAC
535
TCGCCTTGCGCTCCAATAAC
536
AAGCAGAATGACATCGTGCC
537
TCGAAGTCGCTGAATCACGC
SELEX Substrate specific (S)
531
AATCGCTTCGTCGTGGCAAC
Phagemid specific primers
F1
GGTGACCGCGTATTATTACC
R1
TTATGCTTCCGGCTCGTATG

F2
CTCACTATAGGGCGAATTGG
R2
CCAGGTATTCAGGTCAATCC
Primers for sequencing
T7
TAATACGACTCACTATAGGG
SP2
ATTTAGGTGACACTATAGAA
